# Genoarchitecture of the Early Postmitotic Pretectum and the Role of Wnt Signaling in Shaping Pretectal Neurochemical Anatomy in Zebrafish

**DOI:** 10.3389/fnana.2022.838567

**Published:** 2022-03-09

**Authors:** Nikola Brożko, Suelen Baggio, Marcin A. Lipiec, Marta Jankowska, Łukasz M. Szewczyk, Michael O. Gabriel, Chaitali Chakraborty, José L. Ferran, Marta B. Wiśniewska

**Affiliations:** ^1^Centre of New Technologies, University of Warsaw, Warsaw, Poland; ^2^Department of Human Anatomy and Psychobiology, School of Medicine, University of Murcia and Institute of Biomedical Research of Murcia -Ű IMIB, Virgen de la Arrixaca University Hospital, Murcia, Spain

**Keywords:** zebrafish, brain, prosomeric model, pretectum, Wnt signaling, LEF1, TCF7L2

## Abstract

The pretectum has a distinct nuclear arrangement and complex neurochemical anatomy. While previous genoarchitectural studies have described rostrocaudal and dorsoventral progenitor domains and subdomains in different species, the relationship between these early partitions and its later derivatives in the mature anatomy is less understood. The signals and transcription factors that control the establishment of pretectal anatomy are practically unknown. We investigated the possibility that some aspects of the development of pretectal divisions are controlled by Wnt signaling, focusing on the transitional stage between neurogenesis and histogenesis in zebrafish. Using several molecular markers and following the prosomeric model, we identified derivatives from each rostrocaudal pretectal progenitor domain and described the localization of *gad1b*-positive GABAergic and *vglut2.2*-positive glutamatergic cell clusters. We also attempted to relate these clusters to pretectal nuclei in the mature brain. Then, we examined the influence of Wnt signaling on the size of neurochemically distinctive pretectal areas, using a chemical inhibitor of the Wnt pathway and the CRISPR/Cas9 approach to knock out genes that encode the Wnt pathway mediators, Lef1 and Tcf7l2. The downregulation of the Wnt pathway led to a decrease in two GABAergic clusters and an expansion of a glutamatergic subregion in the maturing pretectum. This revealed an instructive role of the Wnt signal in the development of the pretectum during neurogenesis. The molecular anatomy presented here improves our understanding of pretectal development during early postmitotic stages and support the hypothesis that Wnt signaling is involved in shaping the neurochemical organization of the pretectum.

## Introduction

The pretectum is a part of the vertebrate brain that integrates visual information and controls oculomotor functions (Kubo et al., 2014; Naumann et al., 2016; Wang et al., 2019). Oculomotor dysfunction is common in schizophrenia (Morita et al., 2020), linking the pretectum to this disorder. The pretectum is also involved in complex behavioral responses to visual signals, providing inhibitory, excitatory, and modulatory inputs to a network of connections in the brain (Yáñez et al., 2018; Antinucci et al., 2019). The role of the pretectum in health and disease is still poorly understood. Research on animal models would increase our knowledge of the physiology and pathology of the pretectum, but this research suffers from the lack of a unified view of the anatomy of the pretectum in different species. The pretectum, like all diencephalic structures, is characterized by a complex nuclear organization. Despite recent efforts to standardize the nomenclature of the pretectal nuclei, they are still named inconsistently in different species (Ferran et al., 2009; Morona et al., 2017; Puelles, 2019). Moreover, it is difficult to relate their homology in different vertebrates because the origin of the different pretectal nuclei from individual progenitor domains is not definitively resolved. Comparative studies in a developmental context are thus necessary.

Genoarchitectonic studies demonstrated that the pretectum originates from the alar plate of the most caudal part of the diencephalon, called prosomere 1 (Ferran et al., 2007, 2009; Puelles, 2019). These studies described evolutionarily conserved rostrocaudal progenitor domains of the pretectal region, named according to their relative positions to the posterior commissure: the precommissural (PcP), the juxtacommissural pretectum (JcP) and the commissural (CoP) pretectum (Redies, 2000; Ferran et al., 2007, 2008; Lauter et al., 2013). The prosomeric model has also been applied to analyze pretectal regionalization during the early postmitotic and later stages of pretectal nuclei formation in *Xenopus laevis*, chicks, and quail, enabling attempts to link the origin of pretectal derivatives from developmental subdomains to the mature nuclei (Ferran et al., 2007, 2009; Merchán et al., 2011; Morona et al., 2011, 2017). Although zebrafish is a popular model in neuroresearch, the development of its pretectum was poorly investigated at this stage, and the relationship between the progenitor domains and the mature pretectal nuclei remains enigmatic.

Equally unknown are the biological mechanisms shaping the development of the pretectum. The pretectum develops far from the diencephalic and mesencephalic organizers, the zona limitans intrathalamica and the isthmus, respectively. Therefore, physiological amounts of Shh and Fgf released from these signaling centers would not suppress the pretectal fate, as it does with adjacent regions (Vieira and Martinez, 2006; Vieira et al., 2010). A part of pretectal development could be directly instructed by canonical Wnt signals secreted by the organizer center of nearby roof plate (Duncan et al., 2015); however, this possibility has not been investigated.

Here, by *in situ* hybridization and following the prosomeric model, we identified pretectal domains and located some clusters of GABAergic and glutamatergic cells in the early postmitotic diencephalon of zebrafish. In zebrafish, the main wave of neurogenesis starts around 24 hpf and is virtually complete at 48 hpf (Muto and Kawakami, 2013; Boulanger-Weill and Sumbre, 2019). Nucleogenesis in the pretectum progresses gradually as the newborn neurons migrate and cluster into distinct nuclei. We focused on this transition period of early histogenesis to establish a link between the previously described pretectal progenitor domains (Lauter et al., 2013) and the cytoarchitectonic pretectal nuclei at later stages (Yáñez et al., 2018). We concluded this part by identifying the main rostrocaudal pretectal domains and some GABAergic and glutamatergic clusters in the zebrafish pretectum. Furthermore, we investigated the role of canonical Wnt signaling in the development of the neurochemical architecture of the pretectum, using chemical inhibition and CRISPR/Cas9-mediated mutagenesis to knock out downstream components of the Wnt pathway—the Lef1 and Tcf7l2 transcription factors. This experiment revealed the role of Wnt signaling in promoting GABAergic and inhibiting glutamatergic development in some areas of the pretectum.

## Materials and Methods

### Zebrafish and Mouse Lines and Care

Strain ABxTL or AB zebrafish (*Danio rerio*) were maintained in recirculating habitats at 28°C on a 14/10 h light-dark cycle. After natural spawning, larvae were collected, incubated at 28°C, and staged at hours post-fertilization (hpf) according to Kimmel et al. (1995). Larvae were grown in fresh E3 medium, which was changed every other day. To avoid pigmentation, the E3 medium was supplemented with 0.2 mM 1-phenyl-2-thiourea (PTU, Sigma Aldrich, St. Louis, United States). Zebrafish larvae were enzymatically dechorionated with 5% pronase solution (Sigma-Aldrich). To collect samples, zebrafish were anaesthetized by hypothermic shock for 10 min.

The C57BL/6N mice used in this study were bred in a temperature-regulated (22 ± 1°C) animal facility with a 12-h light-dark cycle and unlimited access to food and water.

All experimental procedures strictly adhered to the European Community (86/609/EEC) and Polish government (Dz.U. 2015 poz. 266) guidelines for ethical experimentation.

### Zebrafish Whole-Mount *in situ* Hybridization

Zebrafish larvae were transferred to 4% paraformaldehyde (PFA, Sigma-Aldrich) in 0.1 M phosphate-buffered solution (PBS) and fixed at room temperature (RT) for 3 h or at 4°C overnight. Subsequently, the fixed larvae were washed twice in PBS containing 0.05% Tween (PBS-Tween), dehydrated in increasing methanol concentrations (25, 50, 75, 2 × 100%) in PBS-Tween, and stored at –20°C. On the day of the staining, fixed larvae were rehydrated in methanol/PBS-Tween with decreasing methanol concentrations. To increase cell membrane permeability, larvae were treated with 1 μl/ml proteinase K for 45 min, fixed again in 4% PFA and washed in PBS-Tween. Larvae were then incubated in standard prehybridization solution (50% formamide, 5 × SSC, 100 μg/ml yeast RNA, 50 μg/ml heparin, 0.125% Tween 20, 0.02 M citric acid, pH 6.0) for 4 h at 68–70°C. Thereafter, the solution was replaced with a fresh one containing 200 ng of a labeled probe (the list of probes—Supplementary Tables 1, 2), and the larvae were incubated overnight at 68–70°C. The next day, larvae were washed 6 times in a hot saline-sodium citrate (SSC) buffer (70°C) with decreasing formamide concentration and twice in PBS-Tween at RT. Endogenous peroxidase activity was blocked by incubating larvae with 2% H_2_O_2_ in PBS-T for 1 h. Then, larvae were then incubated in Tris-buffered saline (TBS) containing 0.05% Tween and 0.5% Perkin Elmer Blocking Reagent (TBS-Tween-B) for 4 h. For probe detection, larvae were incubated overnight at 4°C with TBS-Tween-B solution containing anti-DIG (1:1,000) or anti-Fluorescein-HRP (1:5,000) conjugates (Perkin Elmer, Waltham, United States). The next day, larvae were washed 8 times in TBS-Tween and once in Amplification Diluent (Perkin Elmer), and then incubated for 1 h in Amplification Diluent containing Tyramide Signal Amplification (PerkinElmer), the Tyr-Cy3 (Tyramide-Cyanine3 conjugate) or Tyr-Fluorescein (Tyramide-Fluorescein conjugate). Next, larvae were washed twice in TBS-Tween, incubated in 2% H_2_O_2_ in PBS-T for 1 h, and finally washed 4 times with PBS-Tween. The stained larvae were visualized under a confocal Zeiss Axio Imager Z2 LSM700, embedded in low melting agarose.

### Zebrafish Brain Sections and Immunofluorescence

Zebrafish larvae were fixed overnight at 4°C in 4% PFA/PBS. The next day, samples were cryoprotected with 30% sucrose and later embedded in Optimal Cutting Temperature (OCT) medium. Frozen samples were sectioned in a Leica CM1860 cryostat (Leica Microsystems, Wetzlar, Germany) into 20 μm thick slices mounted on glass SuperFrost Plus slides (Thermo Fisher Scientific, Waltham, United States). The slides were stored at –20°C. On the day of staining, the sections were brought to RT, washed three times in PBS and once in PBS-Tween. Then the sections were incubated with 1% bovine serum albumin and 2% normal donkey serum in PBS-Tween overnight. The next day, the sections were washed in PBS and primary antibodies (Supplementary Table 3) were applied overnight at 4°C. Next, slides were washed in PBS-Tween and incubated with secondary antibodies for 1 h at RT. Finally, slides were washed in PBS and sealed with coverslips in Fluoromount with DAPI to stain cell nuclei. The stained slides were stored at –20°C or visualized under a Zeiss Axio Imager Z2 LSM700 confocal microscope.

### Volume Quantification of Brain Structures

The volume of brain structures was measured using Imaris software (Microscopy Image Analysis Software, Oxford Instruments, Abingdon, United Kingdom). 3D models of zebrafish brain structures were computed from 3D images (confocal Z-stack) using the segmentation function. For each structure, the volume was calculated using the 3D renderings.

### IWR-1 Treatment

IWR-1 was dissolved in dimethyl sulfoxide (DMSO) and stored at -20°C. Zebrafish larvae were raised in E3 medium with 0.5% DMSO and 40μg/ml IWR-1 at 28°C for the time indicated. Zebrafish were then fixed in 4% PFA/PBS.

### Reverse Transcription Quantitative Polymerase Chain Reaction

48 hpf control and IWR-1 treated larvae were lysed in RLT buffer (Qiagen, Hilden, Germany) supplemented with 1% β-mercaptoethanol (Sigma-Aldrich). The lysates were homogenized manually with disposable pestles in Eppendorf tubes, and then additionally homogenized in QIAShreder spin columns (Qiagen). RNA was isolated with Rneasy Plus Mini Kit (Qiagen) and eluted with MiliQ water (Millipore). RNA quality and concentration were determined using DS-11 spectrophotometer (DeNovix, Wilmington, United States). cDNA was synthesized from 500 ng RNA with Transcriptor High Fidelity cDNA Synthesis kit (Roche, Basel, Switzerland). RT-qPCR was performed in a LightCycler480 (Roche) on cDNA synthesized from 20 ng RNA per point, using primers in the final concentration of 0.5 nM and SYBR Green chemistry (Applied Biosystems, Waltham, United States). The following primers were used: *axin2* forward ggacacttcaaggaacaactac and reverse cctcatacattggcagaactg (Yin et al., 2012), *gapdh* forward cgctggcatctccctcaa and reverse cagcaacacgatggctgtag (Tang et al., 2007). The relative levels of the amplified transcripts were analyzed with the 2^ddCT (two to the power of delta-delta cycle threshold) method.

### CRISPR/Cas9-Mediated Gene Knockout

Genes were edited using the CRISPR/Cas9 technique with crRNA/tracrRNA guiding approach from Integrated DNA Technologies (IDT),^1^ according to the manufacturer’s protocol.

The crRNA/tracrRNA/Cas9 method proved to efficiently knockout genes in F0 zebrafish (Hoshijima et al., 2019). crRNA guide sequences were designed using IDT’s online tool to target β-catenin-binding domain in *lef1* (GAACGGGATCATTTCATCGG) and *tcf7l2* (GTTCAAGAGCCCACCGTACC) or the *lacZ* gene as a control. Individual crRNAs were hybridized with Alt-R^®^ tracrRNA molecules (IDT, 1072534), and mixed with Alt-R^®^ S.p. HiFi Cas9 Nuclease V3 (IDT, 1081060) to form ribonucleoprotein complexes, which were then injected into zebrafish zygotes.

### Mouse Brain Sectioning and Immunostaining

To obtain the brains, pregnant C57BL/6N mice with embryos at embryonic day (E) 16.5 were sacrificed by cervical dislocation and the embryos were excised and decapitated. The brains were dissected and fixed overnight at 4°C in 4% PFA. They were then incubated in 30% sucrose solution in PBS for 3 days. To prepare tissue blocks, brains were embedded in a solution of 10% gelatine and 10% sucrose in PBS and frozen in –40°C isopentane. Samples were cut into 20 μm thick sections using a Leica CM1860 cryostat (Leica Microsystems) and sagittal sections were collected on SuperFrost-plus glass slides. To stain the tissue sections, the slides were washed three times for 5 min each in 0.2% Triton in PBS (PBS-Triton), with the first wash in warm (37–40°C) PBS-Triton. Antigen recovery was performed by incubating the slides for 30 min at 95°C in citrate buffer solution. They were then washed once for 5 min in PBS-Triton. Blocking was performed by incubation in 5% normal donkey serum (NDS) containing 0.3 M glycine in PBS for 1 h at RT. The tissue was stained overnight at 4°C with the primary antibodies: rabbit anti-TCF7L2 (C48H11, 1:1,000, Cell Signaling, Danvers, United States), rabbit anti-PAX6 (901301, 1:200, BioLegend, San Diego, United States), and mouse anti-GAD67 (MAB5406, 1:500, Merck Millipore, Burlington, United States). The next day, the sections were washed three times in PBS-T for 10 min each and stained with appropriate secondary antibodies conjugated to Alexa Fluor 488 or 594 from Thermo Fisher Scientific (1:500) for 1 h at room temperature. Finally, the slides were washed three times in PBS-Triton for 10 min each with gentle rocking. Mounting was performed in Fluoromount-GTM with Dapi (00-4959-52, Thermo Fisher Scientific) and a Nikon Eclipse Ni-U microscope was used to capture the images.

### Statistical Analysis

Data obtained from quantification of the volume of brain structures and RT-qPCR (ddCT values) were analyzed by one-way ANOVA followed by Dunnett’s *post hoc* test. Data from cell number counting at two anatomical levels were analyzed by a two-tailed unpaired *T*-test. In all cases, the normal distribution of the data was confirmed by D’Agostino-Pearson test and Shapiro-Wilk tests. The scatter plots depict the number of biological replicates analyzed in each group. Statistical significance was presented as follows: *p < 0.05, ^**^p < 0.01, ^***^p < 0.001 and ^****^p < 0.0001.

## Results

### Molecular Boundaries of the Pretectum

To investigate the development of the zebrafish pretectum after neurogenesis, we generated a molecular reference map of the diencephalon at 48 hpf. Firstly, we identified the boundaries of the pretectum with genetic markers previously used to describe alar partitions of prosomere 1 (pretectum) and prosomere 2 (caudal thalamus, rostral thalamus and habenula) in the pre-neurogenesis or post-neurogenesis stages in zebrafish.

**FIGURE 1:**
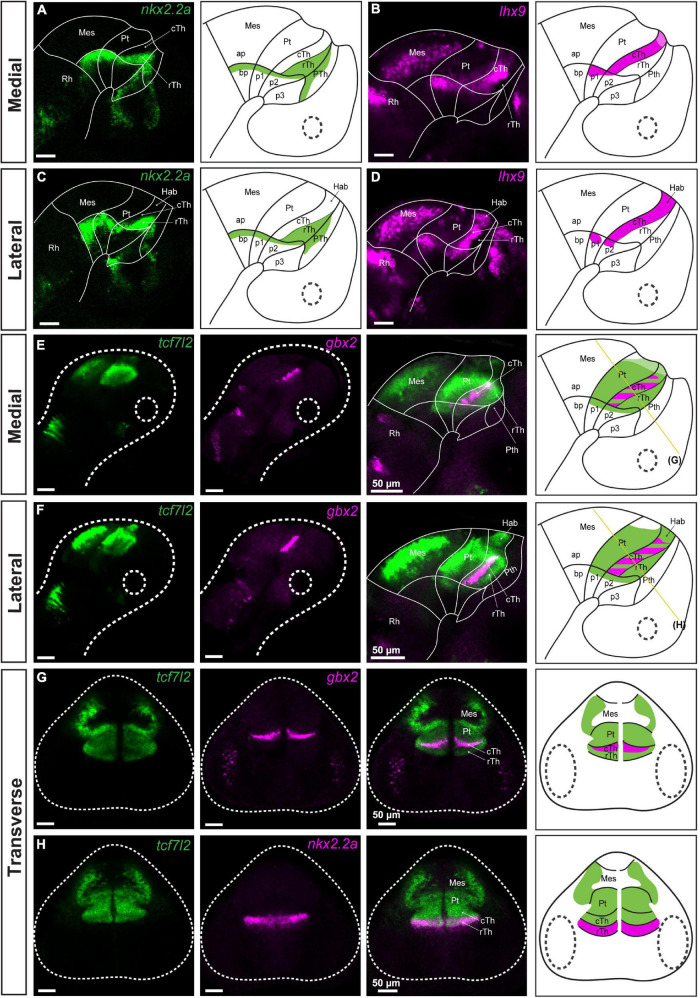
Identification of pretectal boundaries in the diencephalon of 48 hpf zebrafish. Confocal Z-stack images showing brain sections stained using *in situ* hybridization with *nkx2.2a*, *lhx9*, *tcf7l2* and *gbx2* probes. **Sagittal sections: (A–D)** Expression of *nkx2.2a* and *lhx9* in medial **(A,B)** and lateral **(C,D)** sections delineate the alar-basal boundary. Also, *nkx2.2a* expression identifies the rTh and Pth, while *lhx9* expression identifies the cTh and Hab. **(E,F)** Expression of *tcf7l2* identifies prosomere 1 and 2; co-staining of *tcf7l2* and *gbx2* identifies the cTh. **Transverse sections:** **(G,H)** *tcf7l2* co-stained with *gbx2* and *nkx2.2a* marks the Pt, Hab, cTh, and rTh. The schemes show the regions identified by the markers in each merged image. Stripe pattern indicates co-expression of two markers; saturated color indicates high expression signal while lighter color indicates low signal. The yellow lines in **(E,F)** show the sectioning plane of the transverse sections in **(G,H)**. Ap, alar plate; bp, basal plate; Hab, habenula; Mes, mesencephalon; p1, prosomere 1; p2, prosomere 2; p3, prosomere 3; Pt, pretectum; Pth, prethalamus; Rh, rhombencephalon; cTh, caudal thalamus; rTh, rostral thalamus.

*nkx2.2a* and *lhx9* markers were used to identify the alar-basal plate boundary at the level of prosomere 1 and 2 (Figures 1A–D). A stripe of *nkx2.2a* expression extended from the alar plate domain identified as the rostral thalamus or shell posterior to the zona limitans intrathalamica, and reached the basal plate of prosomere 2 by crossing the alar-basal boundary. In addition, this stripe extended caudally toward the basal plate of prosomere 1, bordering the ventral edge of the pretectum (Figures 1A,C), as previously described in chick, zebrafish and *Xenopus laevis* (Ferran et al., 2007; Domínguez et al., 2011; Morona et al., 2011; Lauter et al., 2013). The alar-basal boundary at the level of prosomere 1 is also marked by the dorsal limit of *lhx9* expression (Figures 1B,D), as previously described in zebrafish and *Xenopus laevis* (Morona et al., 2011; Lauter et al., 2013). The localization of the border was confirmed by co-staining of *nkx2.2a* and *lhx9* (Supplementary Figures 1A,B; 72 hpf). *lhx9* was also highly expressed in the caudal thalamus and habenula (Peukert et al., 2011), marking the border between prosomere 1 and 2 in the alar plate (Figures 1B,D).

**FIGURE 2:**
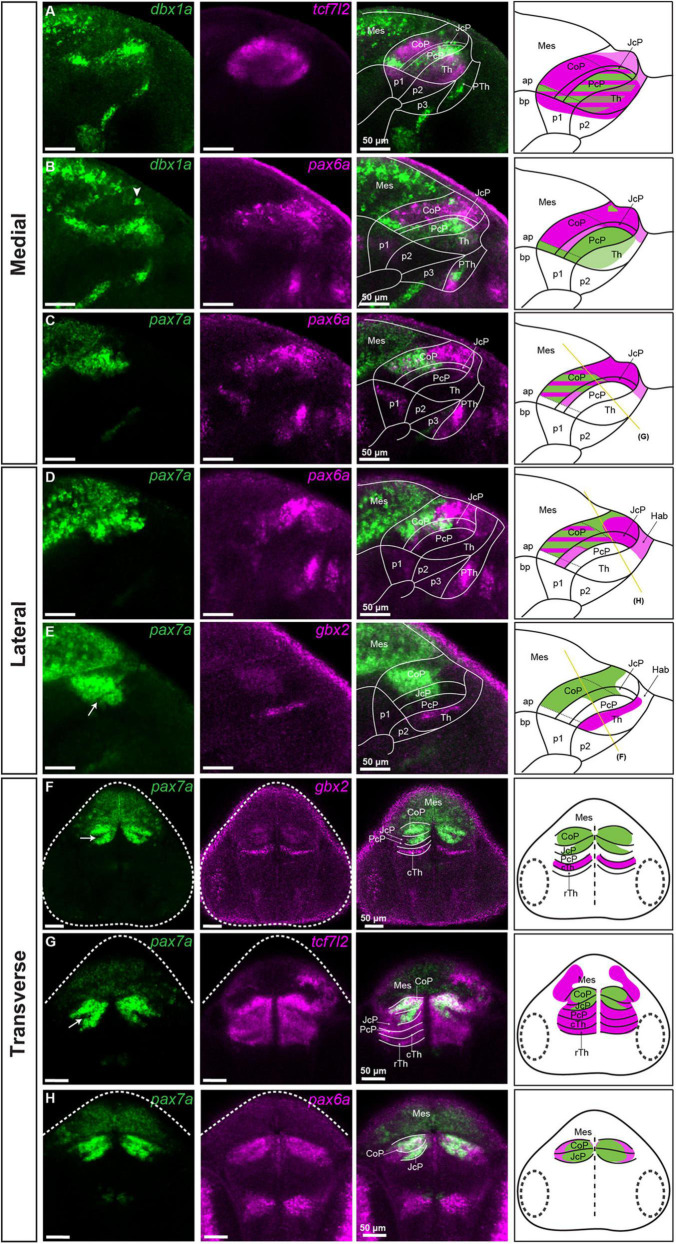
Subdivisions of the pretectum in the brain of 48 hpf zebrafish. Confocal Z-stack images showing brain sections stained using *in situ* hybridization with *dbx1a*, *pax6a*, *pax7a*, *gbx2* and *tcf7l2* probes. **Sagittal sections: (A)** *dbx1a* expression identifies the PcP in the area of *tcf7l2* expression. **(B)** Co-staining of *dbx1a* and *pax6a* shows the alar-basal boundary in prosomere 1 and identifies the *pax6a*-positive area as the CoP and JcP. **(C,D)** Co-staining of *pax7a* and *pax6a* differentiates between the dorsal, central, and ventral subdomains in the Pt. **(E)** Staining of *pax7a* identifies the CoP and JcP and staining of *gbx2* identifies cTh in the lateral section. **Transverse sections: (F–H)** *pax7a* and *gbx2* identify the pretectal domains and the cTh **(F)**, while *tcf7l2* stains the Pt and Th **(G)**, and *pax6a* identifies the CoP and JcP **(H)**. The schemes show the regions identified by the markers in each merged image, with the Pt divided into three rostrocaudal domains: PcP, JcP, and CoP. Stripe pattern indicates co-expression of two markers; saturated color indicates high expression signal while lighter color indicates low signal. The yellow lines **(C–E)** show the sectioning plane of the transverse sections **(F–H)**. The white arrowhead shows the *dbx1a* cluster in the dorsal pretectum **(B)**. The white arrow shows the *pax7*-negative area between the CoP and JcP. ap, alar plate; bp, basal plate; Hab, habenula; Mes, mesencephalon; p1, prosomere 1; p2, prosomere 2; p3, prosomere 3; Pt, pretectum; PcP, precommissural pretectum; JcP, juxtacommissural pretectum; CoP, commissural pretectum; Pth, prethalamus; Th, thalamus; cTh, caudal thalamus; rTh, rostral thalamus; Rh, rhombencephalon.

We analyzed *tcf7l2* as a marker of prosomere 1 and 2 alar plates to molecularly define the entire alar plate domain of prosomeres 1 and 2 between 48 and 72 hpf (Ferran et al., 2007; Merchán et al., 2011; Morona et al., 2011; Lauter et al., 2013). Unexpectedly, the expression of *tcf7l2* extended into the basal plate of prosomeres 1 and 2, confirmed by co-staining with *gbx2* (Figures 1E–G), *nkx2.2a* (Figure 1H) and *lhx2* (Supplementary Figures 1C; **72 hpf)** probes. This pattern of TCF7L2 expression in the basal plate was similar to the mouse embryo at the corresponding age (embryonic day 16.6, E16.5) (Supplementary Figure 2). The expression of *tcf7l2* was also analyzed to determine its relation to the rostral thalamic and caudal pretectal boundaries. *tcf7l2* expression stopped rostrally in the thalamo-prethalamic boundary and caudally in the caudal pretectal boundary (Figures 1E,F, 2A). *tcf7l2* on the pretectal side was expressed in the mantle and periventricular strata, but on the midbrain side, it was only observed in the mantle strata (Figures 1G,H). *Tcf7l2* signal was high in the pretectum, except for its dorsal part where the signal was low (Figure 1E, 2A).

The caudal pretectal border was also identified at this stage by the expression of *pax6a*, which stoped at this limit, and the expression of *pax7a*, being very strong on the pretectal side compared to the midbrain side (Figures 2B–E). The rostral pretectal border was identified by co-staining of *tcf7l2* with *gbx2* (a marker of the caudal thalamus in prosomere 2; Kikuta et al., 2003; Peukert et al., 2011), indicating the pretecto-thalamic boundary (Figures 1E–H). The pretecto-thalamic boundary was also identified by the rostral border of the high expression of *dbx1a* observed on the pretectal side (Figures 2A,B).

Finally, the habenula, a derivative of the dorsal alar plate of prosomere 2, was identified as an *lhx9*/*pax6a*/*tcf7l2*-positive area (Beretta et al., 2013; Lauter et al., 2013) dorsal to the *gbx2*-positive stripe (Figures 1D,F) or as a *pou4f1* (alias *brn3a*)-positive region (Supplementary Figure 3; Roussigné et al., 2012). Additionally, co-staining of *lhx9* and *pou4f1* revealed an asymmetric expression of *lhx9* that has not been previously reported in the habenula (Supplementary Figure 3).

The staining that delineated the pretectum and identified the adjacent structures showed that the pretectum in 48–72 hpf zebrafish is much larger than the caudal thalamus, as was observed in early development in chicken and *Xenopus laevis* (Ferran et al., 2007; Morona et al., 2011, 2017); but in contrast to the relative size of these structures in mammals (Ferran et al., 2008).

### Subdivisions of the Pretectum

To identify the three rostrocaudal pretectal domains defined as commissural, juxtacommissural and precommissural in the postmitotic pretectum, we analyzed the spatial expression patterns of *pax6a, pax7a*, and *dbx1a*, previously described in zebrafish prosomere 1 at the pre-neurogenesis stage (Lauter et al., 2013). These markers were also used in genoarchitectonic studies to describe molecular divisions in the pre-and post-neurogenesis pretectum in *Xenopus laevis*, chicks and quails (Ferran et al., 2007, 2009; Merchán et al., 2011; Morona et al., 2011; Bandín et al., 2015). We noticed that the expression of *dbx1a* was restricted to the ventricular layer at 48 hpf, making this marker suitable only in sections close to the midline at this stage (Figures 2A,B).

The rostrally located precommissural domain (PcP) was identified at the ventricular level as a *dbx1a*-positive thick stripe, but a low-level expression of *dbx1a* was also detected in the caudal thalamus (Figures 2A,B). We confirmed the localization of the PcP in the intermediate strata by co-staining of *pax7a* and *gbx2* in sagittal and transverse sections (Figures 2E,F), where the PcP was visible as a stainless stripe in the most rostral part of the pretectum between the thalamic *gbx2*-positive area and the *pax7-*positive area of the JcP (see below).

The juxtacommissural domain (JcP) located caudally to the PcP was identified in sagittal sections at the level of the ventricular stratum as a *dbx1a*-negative area, except for the most ventral subdomain close to the basal plate, which had a high level of expression (Figures 2A,B). In the *tcf7l2-*positive JcP domain, *pax6a*-positive cells were present in the dorsal and central subdomains of the periventricular layer, and the dorsal subdomain in the mantle zone (Figures 2B–D,H). Cells positive for *pax7a* in the JcP domain were also observed in the periventricular and intermediate strata but were absent in the superficial stratum within the central subdomain (Figures 2C–H). A stainless string in the *pax7a*-positive area tentatively represented a border between the JcP and CoP domains (Arrow; Figures 2E–G).

The caudally located commissural domain (CoP) was identified by low ventricular expression of *dbx1a*, but few cells with increased expression could be seen in the dorsal subdomains (Figures 2A,B). As was the case in the JcP domain, a ventral subdomain with high ventricular expression of *dbx1a* was also observed in the CoP. The *pax6a* mRNA at 48 hpf was highly expressed in the periventricular stratum of the CoP, except for the most ventral subdomain (Figures 2B–D,H). This expression, as was mentioned, extended to the most dorsal subdomains of the JcP and stopped caudally at the midbrain-pretectal boundary. *pax6a* expression was also detected in the inner intermediate and periventricular strata of the CoP, but the highest expression seemed to be in the periventricular layer (Figure 2H). The expression of *pax7a*, which was previously identified in the CoP by birds and *Xenopus laevis* studies (Ferran et al., 2007; Merchán et al., 2011; Morona et al., 2011) and in 28 hpf zebrafish (Lauter et al., 2013), was also detected in the periventricular stratum and throughout the CoP mantle zones of 48 hpf zebrafish (Figures 2C–H). However, the expression was low in the superficial stratum of the CoP, severely reduced in the dorsal area, and absent in the ventral area. Based on the differential expression of *dbx1a*, *pax6a*, *pax7a*, and *tcf7l2*, we delineated dorsal, central, and ventral subdomains of the CoP. The dorsal subdomain of the CoP had few cells expressing *dbx1a*, a high expression of *pax6a* and a weak expression of *pax7a* and *tcf7l2* (Figures 2A–E). The central subdomain of the CoP showed strong expression of *pax7a* and *tcf7l2* in periventricular and mantle zone strata, but a weak or absent expression of *pax6a* in the outer intermediate and superficial strata (Figures 2G,H). Ventral CoP was *pax6a*/*pax7a* negative with strong expression of *dbx1a* and *tcf7l2* (Figures 2A–E).

In summary, the rostrocaudal subdivisions of the pretectum, i.e., PcP, JcP, and CoP, are still molecularly identifiable at 48 hpf, and they can be further subdivided dorsoventrally.

### GABAergic and Glutamatergic Clusters

We then localized clusters of GABAergic and glutamatergic cells in the pretectal area of 48 hpf zebrafish, by co-staining a GABAergic marker, *gad1b* with *gbx2* and *pax6a* or with a glutamatergic marker *vglut2.2* (gene symbol *slc17a6a*). The *gad1b* signal also identified some dopaminergic neurons, because all dopaminergic neurons in the zebrafish pretectum co-release GABA (Filippi et al., 2014).

**FIGURE 3:**
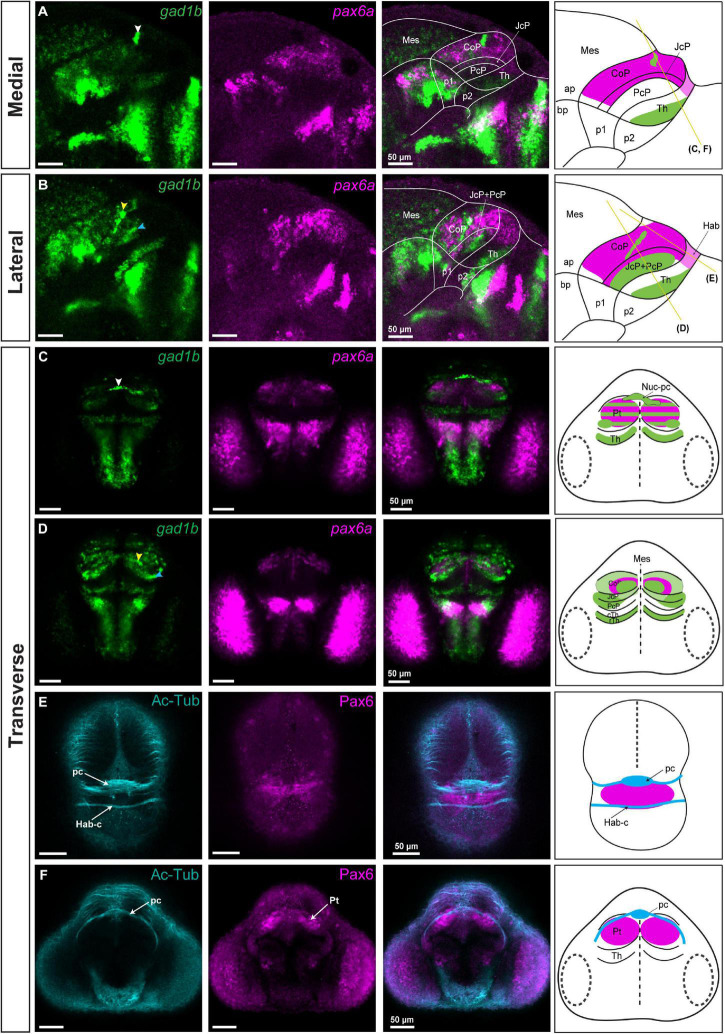
*gad1b*-positive GABAergic clusters and the identification of Nuc-pc in relation to commissures in the diencephalon of 48 hpf zebrafish. Images showing brain sections stained using immunofluorescence with antibodies specific for acetylated-tubulin and Pax6 (Pax6a and Pax6b), and *in situ* hybridization with *gad1b* and *pax6a* probes. **(A,B)** In sagittal sections, *gad1b* and *pax6a* staining identifies a *gad1b*-high GABAergic cluster in the dorsal CoP (**A**, medial section) and two *gad1b*-high clusters in the central subdomain of the rostral Pt (PcP and JcP) and CoP (**B**, lateral section). **(C)** In dorsal sections, co-staining of *pax6a* and *gad1b* identifies the *gad1b*-high cluster as the posterior commissure associated Nuc-pc. **(D)** In ventral sections, co-staining of *pax6a* and *gad1b* identifies a periventricular *gad1b*-high cluster in the CoP and a lateral *gad1b*-high cluster in the rostral Pt. **(E,F)** Acetylated tubulin and Pax6-positive staining identifies the habenular commissure **(E)** and posterior commissure **(F)** in transverse sections. White, yellow and blue arrowheads indicate the dorsocaudal, periventricular and lateral *gad1b* GABAergic clusters, respectively. The schemes depict *gad1b*-high clusters and the regions identified by the markers in each merged image. Yellow lines show the sectioning plane of the transverse sections. The stripe pattern indicates a co-expression of two markers in a region. ap, alar plate; bp, basal plate; Hab, habenula; Hab-c, habenular commissure; Mes, mesencephalon; Nuc-pc, nucleus of the posterior commissure; pc, posterior commissure; Pt, pretectum; CoP, commissural pretectum; JcP, juxtacommissural pretectum; PcP, precommissural pretectum; p1, prosomere 1; p2, prosomere 2; p3, prosomere 3; Th, thalamus; cTh, caudal thalamus; rTh, rostral thalamus; Pt, pretectum.

*Gad1b* signal was detected throughout the CoP, except for its dorsal subdomain (Figures 3A,B). Within the CoP, we observed clusters with particularly high *gad1b* signals. One cluster, located caudodorsally, crossed the midline and extended bilaterally into the superficial stratum of the dorsal CoP (white arrowhead; Figures 3A,C). Another cluster in the periventricular stratum of the CoP extended into the intermediate strata of the CoP at a distance from the JcP (yellow arrowhead, Figures 3B,D). Strikingly, both *gad1b* clusters in the CoP were *pax6a*-negative.

JcP was predominantly GABAergic, had low *gad1b* signal in the periventricular stratum, and a *gad1b*-high cluster in the outer intermediate and superficial strata in the central and ventral subdomains (Figures 3A,B). This lateral GABAergic cluster seemed to overlap partly with the superficial strata of the PcP domain (blue arrowhead; Figures 3B,D).

In an attempt to relate *gad1b*-high clusters to anatomical correlates, we visualized axons and localized them in relation to the Pax6a/b-positive area in the pretectum, using immunostaining with anti-acetylated tubulin and anti-Pax6a/b antibodies (Figures 3E,F). Given the location of the caudodorsal GABAergic cluster in the CoP above the posterior commissure, we concluded that this region is part of the posterior commissural nucleus (Nuc-pc) (Chitnis and Kuwada, 1990; Watson et al., 2011).

**FIGURE 4:**
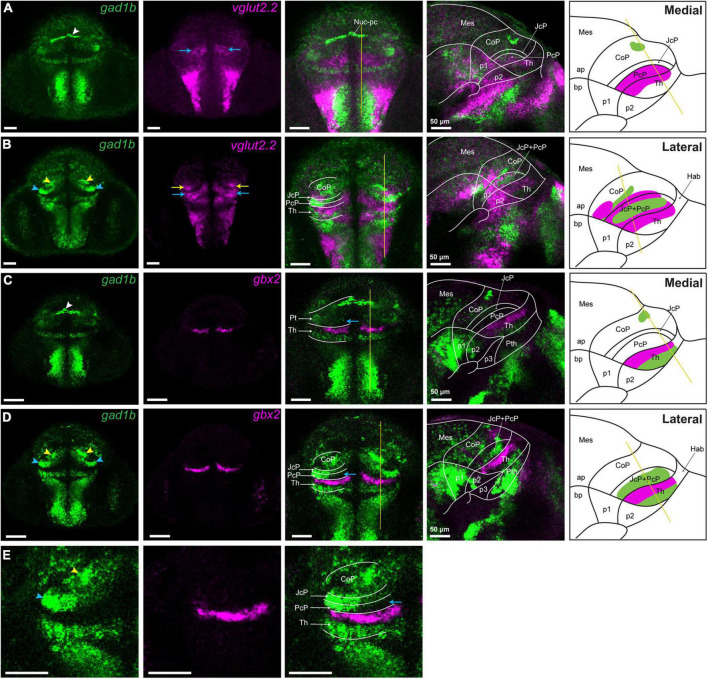
*vglut2.2*-positive glutamatergic clusters in the pretectum of 48 hpf zebrafish. Confocal Z-stack images showing brain sections stained using *in situ* hybridization with *gad1b,vglut2.2* and *gbx2* probes. **(A,B)** Co-staining of *gad1b* and *vglut2.2* identifies *vglut2.2*-positive glutamatergic areas in the diencephalon. Low *gad1b* signal is present throughout the central domain of CoP. **(C,D)** Co-staining of *gad1b* and *gbx2* identifies the cTh and rTh by the *gad1b* expression. **(E)** Magnification of *gad1b* and *gbx2* ventral sections shows the boundary between Pt and Th. White, yellow and blue arrowheads indicate the dorsocaudal, periventricular and lateral *gad1b* GABAergic clusters, respectively. Yellow and blue arrows indicate *vglut2.2* positive glutamatergic clusters in the CoP and PcP, respectively. The schemes depict the regions identified by the markers in each merged sagittal image. ap, alar plate; bp, basal plate; Hab, habenula; Mes, mesencephalon; Nuc-pc, nucleus of posterior commissure; Pt, pretectum; CoP, commissural pretectum; JcP, juxtacommissural pretectum; PcP, precommissural pretectum; p1; prosomere 1; p2, prosomere 2; p3, prosomere 3; Th, thalamus, cTh, caudal thalamus, rTh, rostral thalamus.

We also observed two gltamatergic *vglut2.2*-high clusters in the pretectum (yellow and blue arrow; Figures 4A,B). PcP, which is best seen in transverse sections and overlaps with *gad1b*-free area caudal to the *gbx2*-positive thalamus was *vglut2.2*-high (blue arrow; Figures 4C–E), except for the aforementioned GABAergic cluster in the outer intermediate and superficial strata (blue arrowhead; Figures 4B,D,E). The other cluster of glutamatergic *vglut2.2*-high cells was observed in the CoP. It was located lateral to the periventricular cluster of *gad1b* (yellow arrow; Figure 4B).

Additionally, we compared the pattern of *pax6a/gad1b* expression in the zebrafish brain with the localization of PAX6-positive cells and GAD1 immunoreactivity in the mouse pretectum (Supplementary Figure 4). The pretectal area in the mouse brain was GAD1-positive, with the highest expression in the CoP, but some GAD1-positive cells were also present in some subdomains of the JcP and PcP, resembling the *gad1b* expression pattern in zebrafish. PAX6 was present in the CoP, similar to zebrafish. There were few PAX6-positive cells in the dorsal alar plate of prosomere 2, close to the pretectum. However, unlike zebrafish, PAX6 was practically absent in the JcP, and there were scattered PAX6-positive cells in the dorsal alar plate of prosomere 2, close to the pretectum. This data indicated that the general genoarchitecture of the developing postmitotic pretectum and the establishment of its neurochemical architecture is highly conserved in vertebrates, but some differences can be observed in terms of the number of cells expressing the analyzed marks mainly in the JcP domain.

In summary, in the CoP, we identified two GABAergic clusters with high *gad1b* signal (one of which was identified as Nuc-pc, and the other one we called periventricular) and a lateral *vglut2.2*-positive glutamatergic cluster. We also confirmed the GABAergic identity of the JcP and the glutamatergic identity of the PcP, with a *gad1b*-high cluster in the outer intermediate and superficial strata of these two domain (we called this cluster lateral). It does not ruled out that other clusters may be identified in the pretectum with probes detecting *gad1a* and *vglut2.1*, paralogs of *gad1b* and *vglut.2.2*, respectively.

We then asked whether the Wnt signaling pathway controls the spatial extent of GABAergic and glutamatergic areas in the developing pretectum.

### Spatiotemporal Expression of the Lef1, Tcf7l2, and β-Catenin Proteins

Firstly, we determined whether downstream components of the canonical Wnt signaling pathway Lef1 and Tcf7l2, are present in the zebrafish pretectum between 24 and 48 hpf, using immunofluorescence. We also analyzed the subcellular localization of β-catenin a marker of Wnt signaling activity. Although the expression patterns of the *lef1* and *tcf7l2* genes were known (Li et al., 2009; Wang et al., 2009; Shimizu et al., 2012; Hüsken et al., 2014), no data were available for the protein level.

**FIGURE 5:**
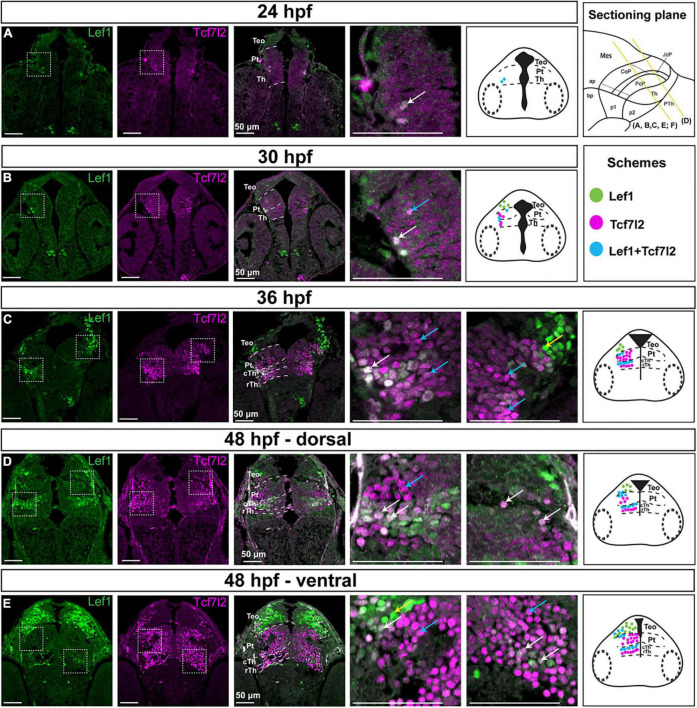
Spatiotemporal expression of the Lef1 and Tcf7l2 proteins in the brain of zebrafish embryos (24–48 hpf). Images showing brain sections from different developmental stages immunostained with antibodies specific for Lef1 and Tcf7l2. **(A) 24 hpf:**: Tcf7l2-positive cells are detected in the area of the diencephalon mantle zone; *lef1* is not expressed in the diencephalon. **(B)** **30 hpf:** more Tcf7l2-positive and few Lef1-positive cells are detected in the area of the diencephalon mantle zone. **(C) 36 hpf:** Lef1-positive cells are visible in the mantle zone in the cTh, caudolateral edge of the Pt and TeO, while Tcf7l2-positive cells are present throughout the Th and Pt with high Tcf7l2 signal in the mantle zone. **(D,E) 48 hpf:** Lef1-positive cells are visible in the cTh and caudolateral edge of the Pt in the dorsal section **(D)**, being less numerous in the cTh in the ventral section **(E)**, Tcf7l2-positive cells are detected throughout the Th and Pt, being more numerous in ventral sections **(E)**. Yellow lines show the sectioning plane of the sagittal sections in **(A–E)**. Blue arrows point to Tcf7l2-positive cells, yellow arrows point to Lef1-positive cells and white arrows point to double-positive Lef1/Tcf7l2 cells in magnified images. The schemes represent the spatiotemporal distribution of Lef1-positive (green dots), Tcf7l2-positive (magenta dots), and double-positive Lef1/Tcf7l2 (blue dots) cells. Pt, pretectum; TeO, optic tectum; Th, thalamus; cTh, caudal thalamus; rTh, rostral thalamus.

At the onset of neurogenesis, 24 hpf, there were only a few Lef1- and Tcf7l2-positive cells in the diencephalon. They were localized in the thin stratum of the mantle zone (Figure 5A). At 30 hpf, sparse Tcf7l2-high cells were present in the mantle zone of prosomeres 1 and 2 (Figure 5B). A weak Tcf7l2 signal was also detected in cells of the periventricular zones compared to intermediate strata of the mantle zone. At 36 hpf, Tcf7l2 levels increased sharply in the expanding mantle zone of the thalamus and pretectum (Figure 5C). Lef1-positive cells occurred in the mantle zone of the caudal thalamus and the most caudal region of the pretectum.

At 48 hpf, in the early postmitotic diencephalon, the number of Tcf7l2-positive cells in the thalamus and pretectum increased. Virtually all cells were Tcf7l2-positive but showing a dorsoventral gradient of signal intensity, with most dorsal subdomains (close to the roof plate) having a low expression (Figures 5D,E) which is consistent with the progress of neurogenesis and cell maturation (Scholpp et al., 2009). In contrast to Tcf7l2, the number of Lef1-positive cells was higher in the dorsorostral part of the thalamus (84 and 57% of Lef1+ cells in the dorsorostral and ventrocaudal parts, respectively, *p* = 0.00022, *n* = 5), suggesting downregulation of Lef1 in more mature cells. The majority (88%) of Lef1-positive cells in the caudal thalamus co-expressed Tcf7l2. In the pretectum, Lef1-positive cells were mainly located in the superficial stratum of the CoP, and they did not express Tcf7l2.

**FIGURE 6:**
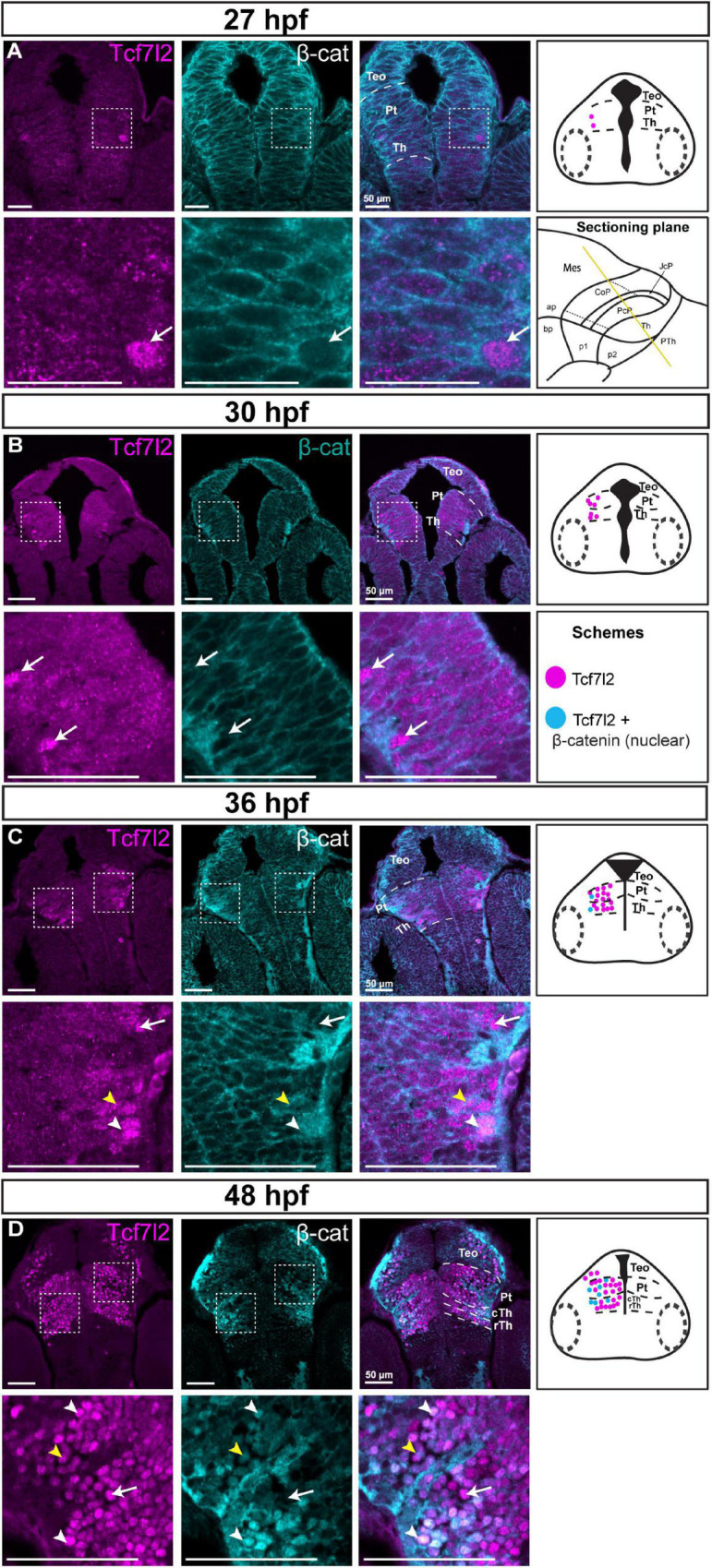
Spatiotemporal expression and subcellular localization of β-catenin in the brain of zebrafish embryos (27–48 hpf). Images showing brain sections from different developmental stages immunostained with antibodies specific for Tcf7l2 and β-catenin. **(A,B) 27 and 30 hpf:** Tcf7l2 is present in a few cells in the diencephalon mantle zone, while the β-catenin protein is localized only in cell membranes throughout the diencephalon. **(C) 36 hpf:** Tcf7l2-positive cells are present throughout the Th and Pt with high Tcf7l2 signal in the mantle zone; β-catenin is localized in cell membranes in the ventricular/periventricular area while its subcellular localization in the diencephalon mantle zone is not clear. **(D) 48 hpf:** Tcf7l2- positive cells are visible throughout the Th and Pt; β-catenin is localized in cell nuclei in the cTh and Pt, being high in superficial areas. Yellow lines show the sectioning plane of the sagittal sections in **(A–D)**. White arrowheads indicate Tcf7l2-positive cells with high level of nuclear β-catenin, yellow arrowheads indicate Tcf7l2-positive cells with low level of nuclear β-catenin, white arrows indicate Tcf7l2-positive cells with no β-catenin in the nuclei. The schemes represent the spatiotemporal distribution of Tcf7l2-positive (magenta dots), and Tcf7l2-positive cells with nuclear β-catenin (blue dots) cells. Pt, pretectum; TeO, optic tectum; Th, thalamus; cTh, caudal thalamus; rTh, rostral thalamus.

At 27 and 30 hpf and in the ventricular/periventricular strata at 36 hpf, β-catenin was detected only in the cell membranes (Figures 6A–C), indicating low activity of Wnt signaling at these stages. The subcellular localization of β-catenin was not clear at the mantle zone at 36 hpf (Figure 6C). Massive accumulation of β-catenin in the nuclei occurred in Tcf7l2-positive cells in the mantle zone of both prosomeres 1 and 2 at 48 hpf (Figure 6D). Thus, the spatiotemporal and subcellular pattern of β-catenin changed with the development of the diencephalon, including the pretectum, and nuclear accumulation of β-catenin followed that of Tcf7l2.

These results revealed high activity of Wnt signaling in postmitotic and maturing pretectal neurons and suggested that Wnt signaling may play a role in the development of the pretectum during neurogenesis.

### Pretectal Phenotypes in Zebrafish With Reduced Activity of Wnt Signaling

To investigate the role of Wnt signaling in establishing the neurochemical anatomy of the pretectum, we used two approaches to inhibit Wnt signaling during neurogenesis. In the first approach, we treated zebrafish larvae with IWR-1 between 24 and 48 hpf. IWR-1 is a cell-permeable small molecule that enhances β-catenin degradation (Huang et al., 2009) and effectively inhibits Wnt signaling in zebrafish larvae (Peukert et al., 2011; Misztal et al., 2017). IWR-1 was added to an E3 raising medium at a concentration of 40 μg/ml. This concentration was sufficient to downregulate *Axin2*, a classical target of the canonical Wnt signaling pathway, which has no major toxic effect after 24 h of incubation (Supplementary Figure 5A). In the second approach, we knocked out *lef1* and *tcf7l2* by acute CRISPR/Cas9-mediated mutagenesis. The efficacy of the knockouts was confirmed in 48 hpf F0 larvae (crispants) at the protein level by immunofluorescence staining of Lef1 and Tcf7l2 in brain sections (Supplementary Figures 5B–D).

To exclude early effects of the treatments, we assessed neurochemical specification of the fate of pretectal progenitors at 30 hpf by *in situ* hybridization with a probe that detects transcripts of a GABAergic pro-neuronal marker *ascl1* (alias *zash1*/*mash1*) and *dbx1a* or *shh* probes. The pretectal progenitor domain was not visibly affected by *lef1* and *tcf7l2* knockout or a 6-h incubation in an IWR-1-supplemented medium (Supplementary Figure 6). This suggests that GABAergic fate specification in the pretectum is not controlled by Wnt signaling, and this is consistent with the absence of Lef1 and Tcf7l2 proteins at this stage.

Next, we investigated the formation of GABAergic and glutamatergic clusters in 48 hpf zebrafish treated with IWR-1 between 24 and 48 hpf or in *lef1*/*tcf7l2* crispant larvae. The samples collected were stained with *gad1b* and *vglut2.2* probes, followed by image acquisition and 3D reconstruction to quantify changes in the volume of cell clusters.

**FIGURE 7:**
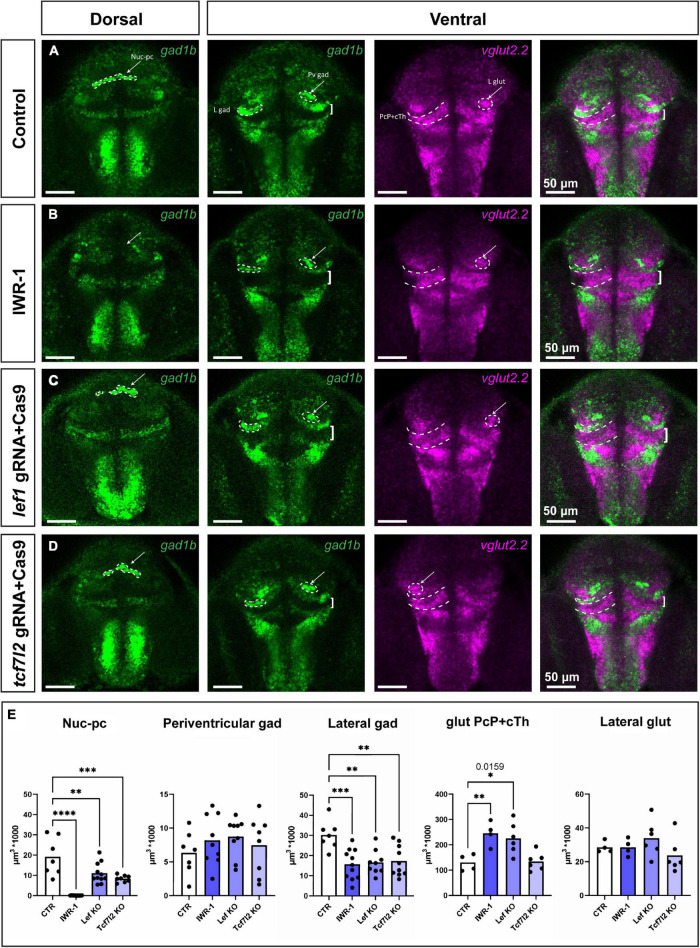
Phenotypes in the pretectum of IWR-1-treated, *lef1* crispant and *tcf7l2* crispant zebrafish at 48 hpf. Confocal Z-stack images showing brain sections stained using *in situ* hybridization with *gad1b* and *vglut2.2* probes to visualize different neurochemical clusters of neurons. **(A) Control:** the *gad1b*-high Nuc-pc is visible in the dorsal section; *gad1b*-high lateral and paraventricular clusters in the ventral section; and *vglut2.2*-positive lateral cluster and *vglut2.2*-positive region of PcP and cTh are visible in the ventral section. **(B) IWR-1:** the Nuc-pc is absent, the lateral *gad1b*-high cluster is smaller, and the *vglut2.2*-positive area of PcP and cTh is bigger. **(C) *lef1* crispants:** The lateral *gad1b*-high cluster is smaller, and the *vglut2.2*-positive area of PcP and cTh is bigger. **(D) *tcf7l2* crispants:** Nuc-pc is smaller and the lateral *gad1b*-high cluster is smaller. **(E)** The graphs show volume quantification of distinct clusters indicated in the previous images. Statistics in **(E)** *n* = 4–10; one-way ANOVA followed by Dunnett’s *post hoc* test; *p < 0.05 (the exact *p*-value is indicated in the graph), ^**^p < 0.01, ^***^p < 0.001 and ^****^p < 0.0001. Nuc-pc, nucleus of the posterior commissure; L gad, lateral *gad1b*-positive cluster; L glut, lateral *vglut2.2*-positive cluster; Pv gad, periventricular *gad1b*-positive cluster; CoP, commissural pretectum; JcP, juxtacommissural pretectum; PcP, precommissural pretectum; cTh, caudal thalamus.

The Nuc-pc was absent in IWR-1-treated larvae and was smaller in *lef1* and *tcf7l2* crispants (Figure 7) than in the control group (one-way ANOVA; [*F*(3, 33) = 22.87; *p* < 0.0001]. The volumes of the periventricular GABAergic and lateral glutamatergic clusters in the CoP were unchanged [*F*(3, 29) = 0.67; *p* = 0.58] and [*F*(3, 16) = 1.37; *p* = 0.29, respectively]. The volume of the *gad1b*-high cluster in PcP (lateral cluster) decreased [*F*(3, 33) = 7.12; *p* = 0.0008]. Finally, the volume of the glutamatergic area that included the caudal thalamus and the PcP increased in the IWR-1-treated and *lef1* crispant groups [*F*(3, 16) = 7.81; *p* = 0.002]. To distinguish between the thalamus and the PcP, samples from IWR-1-treated larvae were stained with *gad1b* and *gbx2* (Supplementary Figure 7). The volume of the *gbx2* caudal thalamus was similar in the control and IWR-1-treated groups, indirectly indicating an enlargement of the PcP in zebrafish larvae with Wnt signaling inhibition or Lef1 deficiency.

These results demonstrated that Wnt signaling plays an instructive role in controlling the size of GABAergic and glutamatergic clusters in the developing zebrafish pretectum. In particular, Wnt signaling is critical for the formation of the Nuc-pc.

## Discussion

Few studies have analyzed in detail the development of progenitor domains in the zebrafish pretectum at 28 hpf (Lauter et al., 2013). However, this zebrafish region is poorly characterized after this stage to the final anatomical organization in the adult. Pioneering studies on the pretectal region of birds and *Xenopus laevis* that cover different stages of development indicate that an adequate characterization in the adult requires an exhaustive interpretation of the processes that occur during the different stages of development (Ferran et al., 2007, 2009; Merchán et al., 2011; Morona et al., 2011, 2017). Here, we studied the development of the pretectum in zebrafish, focusing on the early post-neurogenesis stage.

**FIGURE 8:**
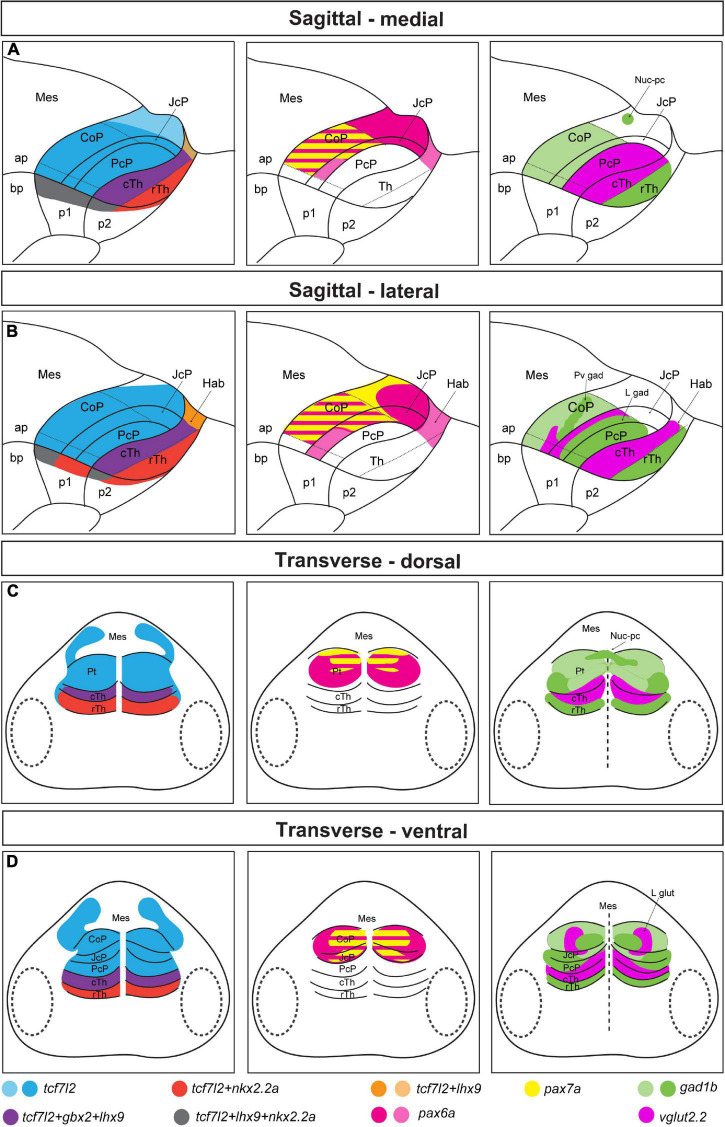
Schematic summary of Figures 1–4. Schemes showing the pattern of marker gene expression and localization of *gad1b* and *vglut2.2*-positive areas in the diencephalon of 48 hpf zebrafish. **(A,B)** Sagittal sections at the medial **(A)** and lateral **(B)** levels. **(C,D)** Transverse section at the dorsal **(C)** and ventral **(D)** levels. In the left panel, *tcf7l2* expression marks the alar plate of prosomere 1 and 2 and extends into the basal plate; *nkx2.2a* and *lhx9* expression delineate the alar-basal plate boundary in prosomere 1 (CoP, JcP, and PcP) and 2 (Hab, cTh, and rTh); *gbx2* and *lhx9* expression in the cTh demarcated the rostral border of the Pt; *nkx2.2a* is also expressed in the rTh, and *lhx9* is also expressed in the habenula. In the middle panel, pretectal domains are patterned by the expression of *pax6a* and *pax7a*. *pax6a* is highly expressed in the dorsal subdomain of the Pt. Both *pax6a* and *pax7a* markers are present in the central subdomains of the CoP and JcP, but their expression decrease rostrally and ventrally. In the right panel, the expression of *gad1b* and *vglut2.2* identifies several GABAergic and glutamatergic clusters: the Nuc-pc in the caudal edge of the CoP dorsal subdomain, periventricular GABAergic and lateral glutamatergic clusters in the central subdomain of the CoP, lateral GABAergic cluster in the area of the JcP and PcP (lateral parts), and glutamatergic PcP (medial parts). Saturated colors indicate high signal and lighter colors low signal of expression. ap, alar plate; bp, basal plate; Hab, habenula; Mes, mesencephalon; Pt, pretectum; p1, prosomere 1; p2, prosomere 2; p3, prosomere 3; Th, thalamus; cTh, caudal thalamus; rTh, rostral thalamus.

In the first part of this study, we used a genoarchitectonic approach (Puelles and Ferran, 2012) and followed the prosomeric model (Nieuwenhuys and Puelles, 2016; Puelles, 2019) as the reference to molecularly identify distinct domains and subdomains in the rostrocaudal and dorsoventral axes of the early postmitotic pretectum in zebrafish (summarized in Figure 8). We found out that the general molecular architecture of the maturing pretectum at 48 hpf is similar to the previously described architecture of prosomere 1 before neurogenesis in zebrafish larvae at 28 hpf (Lauter et al., 2013). At 48 hpf, the three rostrocaudal domains precommissural, juxtacommissural, and commissural of the pretectum can be molecularly recognized. However, the borders between these domains are not as clear as in earlier stages because the number of postmitotic cells is increased in the mantle layer of each anteroposterior pretectal domain. In the stage analyzed here, the key genetic markers whose differential expressions enable the recognition of the main pretectal and intrapretectal limits in previous stages are downregulated. The expression of *dbx1a*, which patterns the caudal thalamus and PcP before neurogenesis (Lauter et al., 2013), is downregulated rostrally and is largely restricted to the ventricular stratum of the PcP and the ventral subdomain of the JcP and CoP in the 48 hpf zebrafish. This is similar to corresponding stages in *Xenopus laevis* (stages 37–42; Morona et al., 2011), chicken and quail [Hamburger–Hamilton stages (HH) 24–28; Ferran et al., 2007; Merchán et al., 2011]. The expression of *pax6a* which is ubiquitous in all pretectal progenitor domains at 28 hpf (Lauter et al., 2013), is not detected in the PcP, gradually decreases in the JcP and ventral subdomains of the CoP, but remains high in the dorsal and central subdomains of the CoP at 48 hpf. A similar restriction of *pax6* during early pretectal development is observed in *Xenopus laevis* and avian embryos (Ferran et al., 2007; Merchán et al., 2011; Morona et al., 2011). *pax7a* continues to be expressed in the central subdomain of the CoP but is scarcely expressed in the outer pretectal layers, where neurons are more mature, and in the dorsal CoP. This suggests a downregulation of the *pax7a* gene in dorsal pretectal neurons and the superficial derivatives of the central subdomain. However, the probes used in the previous study on 28 hpf zebrafish (Lauter et al., 2013) and the current study detected different isoforms produced by this gene [*(pax7a(a)* and *pax7a(c)*, respectively, Seo et al., 1998]. Another possibility is that different *pax7a* isoforms are expressed in different regions of the pretectum.

Small differences also exist in the post-neurogenesis pretectum between zebrafish, *Xenopus laevis* and birds, implying that pretectal development at this stage is similar but not fully conserved between vertebrates. Unlike in chicken and quail, where *pax7* is expressed exclusively in the CoP (Ferran et al., 2007, 2009; Merchán et al., 2011), in *Xenopus laevis* (Morona et al., 2011) and zebrafish, *pax7a* is also expressed in the JcP. Furthermore, a peculiarity of the zebrafish is that *pax6a* is highly expressed in the dorsal part of the CoP at 48 hpf, whereas the expression of *pax7a* is low or absent. However, other isoforms of *pax7a* should be investigated in zebrafish to verify this conclusion.

Previous research assigned the pretectal nuclei to three original progenitor domains using the genoarchitectonic approach and by benchmarking mature pretectal anatomy to the post-neurogenesis stage and a series of intermediate stages in birds and *Xenopus laevis* (Ferran et al., 2009; Morona et al., 2017). Our analysis shows that the neurochemical anatomy of the pretectum is evolutionarily conserved, with the PcP being primarily glutamatergic with a presence of GABAergic cells in outer intermediate and superficial strata (summarized in Figure 8). The JcP highlighted their *gad1b* content, and the CoP showed clusters of neurons of both *gad1b* and *vglut2.2* identities in zebrafish as was observed in previously analyzed *Xenopus laevis* and mice (Morona et al., 2011, 2017; Virolainen et al., 2012). The clusters of strongly immunoreactive cells extended dorsoventrally, as previously described for 48 hpf zebrafish and 4 dpf *Gasterosteus aculeatus* (Ekström and Ohlin, 1995; Mueller et al., 2006). Below, we attempt to interpret the identified clusters in relation to pretectal nuclei in the mature brain defined by cytoarchitectonic and hodological criteria. However, it is necessary to emphasize that the identity for each cluster in relation to the adult anatomy postulated here is a proposal. This is because the 48 hpf stage is an early transition stage in which we do not know if the position acquired by each cluster will be the final one in the adult. For this comparative analysis we use available data on the localization of GABAergic, glutamatergic and dopaminergic [tyrosine hydroxylase (TH) -positive)] regions in the larval (48–72 hpf), juvenile and adult zebrafish pretectum (Mueller et al., 2006; Mueller and Guo, 2009; Filippi et al., 2014; Yáñez et al., 2018; Baier and Wullimann, 2021).

In the commissural pretectum, we identified two *gad1b*-high clusters and one *vglut2.2* cluster. The caudodorsal *gad1b* cluster is located superficial to the posterior commissure. It extends dorsoventrally from the midline as a transverse stripe at each side of the pretectum. A cluster in this localization was immunodetected in 48 hpf zebrafish and interpreted as GABAergic fibers (Mueller et al., 2006). Because our detection is based on *in situ* hybridization which stains cell bodies, we conclude that it contains *gad1b* neurons located along the fibers. Based on the localization of the cluster in relation to the posterior commissure, we interpret it as the primordium of the nucleus of the posterior commissure in the adult, previously described in zebrafish, mice and humans (Chitnis and Kuwada, 1990; Watson et al., 2011). The second *gad1b*-high column is localized in the intermediate stratum in the central subdomain. Similar to the previous cluster, it extends as a transverse column in the dorsoventral direction. In our interpretation, this cluster is a candidate to be part of the periventricular nucleus described in the adult brain based on its neurochemical identity, shape and characteristic stratum localization. This nucleus comprises GABA-co-secreting TH-positive (dopaminergic) cells, previously described in several studies and ambiguously assigned either to the dorsal or ventral part of the periventricular nucleus (Ma, 2003; Mueller and Guo, 2009; Filippi et al., 2014; Yáñez et al., 2018) and identified as the retinorecipient arborization field AF9 (Baier and Wullimann, 2021).

Laterally adjacent to the periventricular *gad1b* cluster of the CoP is the *vglut2.2-*positive cluster. A glutamatergic cluster has been previously described in this localization in the juvenile zebrafish brain but has not been assigned to a specific nucleus (Filippi et al., 2014). Based on its localization, this mass of cells could be interpreted as part of the periventricular or intercalated pretectal nucleus (Yáñez et al., 2018). Finally, the juxtacommissural pretectum is a *gad1b* area. A cluster of cells with *gad1b*-high signal, which is located close to the JcP but in the area of the PcP is likely a mass of cells migrating tangentially from the JcP. A cluster of GABAergic TH-negative cells has previously been identified in a similar location, immediately adjacent to the TH-positive GABAergic cluster of the periventricular nucleus (Filippi et al., 2014), so, it is likely that precommissural cells contribute to this cluster. Finally, the localization of the glutamatergic PcP resembles the localization of the dorsal posterior thalamic nucleus (DP) in the adult brain (Mueller, 2012; Baier and Wullimann, 2021). We speculate that the DP cells could be derived from the caudal thalamus and PcP or may exclusively be part of a PcP derivative. However, since 48 hpf is a period in which the definitive differentiation of the derivatives of each pretectal domain has not occurred, it must be taken into account that it is a transitional state toward the adult and that the assignment of the definitive identity in relation to that of the adult pretectum will require an analysis of the successive stages of development. Future studies including fate maps are required to establish the precise correlation of each derivative in the successive stages of development with the anatomy observed in the adult.

The relatively small size of the thalamus compared to that of the pretectum at the 48 hpf stage of zebrafish was observed in the early pretectal characterization of chicken, quail and *Xenopus laevis* (Ferran et al., 2007, 2008; Merchán et al., 2011; Morona et al., 2011). This reduced periventricular thalamic territory observed in early stages is maintained in later stages of *Xenopus laevis* but is expanded in the outer mantle zone (see Figures 8J–L, 12M–P in Morona et al., 2017). However, visual observation of the relative size of the mouse thalamus obtained from molecular maps that recognize the periventricular region of the thalamus suggests that its size at E11.5 would be similar to that of the pretectum, but at E12.5, it exceeds the pretectal region in relative proportion (see Ferran et al., 2008).^2^ Further comparison will be required in later stages of the zebrafish to determine whether the periventricular extension of the thalamus is enlarged compared to the pretectal region.

The pretectum, being a part of the diencephalon, develops in an area of high Wnt signaling activity previously visualized in reporter fish (Shimizu et al., 2012). We detected unambiguous Wnt activity in the mantle zone of the dorsal diencephalon as attested by the nuclear localization of β-catenin and the appearance of Tcf7l2 and Lef1 proteins, following much earlier expression of the *lef1* and *tcf7l2* genes in progenitor cells (Shimizu et al., 2012). During neurogenesis, Tcf7l2 and nuclear β-catenin are present in cells of the pretectal mantle zone, and there are some Lef1-positive cells in its caudal part and the caudal thalamus bordering the rostral part of the pretectum. This timing of the Wnt pathway mediators’ occurrence in the thalamus and pretectum is consistent with the activity of Wnt signaling detected in Wnt reporter lines (Dorsky et al., 2002; Shimizu et al., 2012). The potential sources of the Wnt signal are the MDO and the roof plate of the diencephalon/mesencephalon which express high levels of at least three canonical Wnt genes *wnt1*, *wnt3*, and *wnt3a* (Peukert et al., 2011; Duncan et al., 2015). However, in postmitotic neurons of the diencephalon, downstream components of the Wnt pathway can be constitutively active even when Wnt signal transduction is inhibited (Misztal et al., 2011). Therefore, Wnt signal-independent mechanisms can contribute to maintaining a high level of nuclear β-catenin in the postmitotic pretectum.

In the final part of this study, we showed that the size of GABAergic and glutamatergic clusters of cells in the pretectum depends on the activity of Wnt signaling (more precisely, on the activity of its intracellular components, β-catenin and Lef1/Tcf7l2) during neurogenesis. The β-catenin signaling pathway appears to play an instructive role in the development of the pretectum during neurogenesis by promoting the growth of the caudodorsal and the precommissural/juxtacommissural GABAergic *gad1b* clusters while inhibiting the expansion of the glutamatergic *vglut2.2*-positive precommissural domain. These functions are likely mediated by Lef1 and Tcf7l2 because the deficiency of either of them and the inhibition of Wnt signaling at the level of β-catenin produced similar effects on GABAergic areas. Lef1 deficiency and Wnt inhibition also affected the size of the glutamatergic area.

Similar phenotypes caused by crispants of *lef1* and *tcf7l2* suggest that Lef1 and Tcf7l2 act synergistically in the development of the neurochemical anatomy of the pretectum. The mechanism is not known but hypothetically, it could involve regulation of cell cycle exit in progenitor cells, cell migration, postmitotic specification of neurochemical identity, or maturation rate. An intriguing question is whether Lef1 and Tcf7l2 act in a cell-autonomous or non-autonomous manner. The latter is consistent with the expression of Lef1 in areas adjacent to the affected clusters, i.e., in the caudal thalamus and caudolateral aspect of the pretectum, but not in the clusters themselves. The cell non-autonomous role of transcription factors in the development of the brain is not unusual. For example, Lhx2 and Lhx9 in postmitotic cells in the caudal thalamus limit the expression of *pcdh10* in progenitor cells and the extension of the *pacdh10*-positive area into the pretectum (Peukert et al., 2011).

The molecular and anatomical development of the intermediate, later, and postnatal stages of development of the pretectal region is poorly characterized in zebrafish, and most of the data available are from birds and *Xenopus laevis* at different stages of maturation. This knowledge gap in zebrafish and other species makes it difficult to characterize the homologies between the pretectal nuclei in vertebrates and hinders research into the functional role of the pretectum and pretectal pathologies that may be associated with impaired development. Our research helps to fill this gap by postulating some links between the progenitor subregions of the pretectum and the pretectal nuclei in the mature brain of zebrafish. However, further works will be necessary to map the fate of each progenitor domain during all the stages of development to the final mature anatomy of the pretectal region. We also recognize the role of Wnt signaling and its mediators Lef1 and Tcf7l2 in the formation of neurochemically distinct clusters, which contributes to the understanding of the development of the pretectum from prosomere 1 to a multinuclear area with diverse neurochemical identity. Further research would investigate the cellular mechanisms underlying this function of Wnt signaling.

## Data Availability Statement

The original contributions presented in the study are included in the article/Supplementary Material, further inquiries can be directed to the corresponding author/s.

## Ethics Statement

Ethical review and approval was not required for the animal study because the study was performed on embronic stages which does not require ethical approvals, in compliance with the current normative standards of the European Community (86/609/EEC) and the Polish Government (Dz.U. 2015 poz. 266).

## Author Contributions

NB and MW conceived and planned the study. NB performed majority of experiments. NB and SB prepared the figures and wrote the first draft of the manuscript. ML designed and prepared guide RNAs for CRISPR/Cas9-mediated gene editing. NB, ŁS, SB, and MW performed 3D analyses and statistics. MJ optimized IWR treatment and performed some *in situ* hybridization stainings. MG performed immunostaining of mouse brain sections and did the proofreading. CC helped to design probes and validate gene editing. NB, SB, JF, and MW interpreted the results. All authors contributed to the article and approved the submitted version.

## Conflict of Interest

The authors declare that the research was conducted in the absence of any commercial or financial relationships that could be construed as a potential conflict of interest.

## Publisher’s Note

All claims expressed in this article are solely those of the authors and do not necessarily represent those of their affiliated organizations, or those of the publisher, the editors and the reviewers. Any product that may be evaluated in this article, or claim that may be made by its manufacturer, is not guaranteed or endorsed by the publisher.

## Footnotes

1
https://eu.idtdna.com

2
http://atlas.brain-map.org/
