# Supplementary material for: Genoarchitecture of the Early Postmitotic Pretectum and the Role of Wnt Signaling in Shaping Pretectal Neurochemical Anatomy in Zebrafish
Source: Front Neuroanat. 2022 Mar 9;16:838567. doi: 10.3389/fnana.2022.838567 (PMC8959918; doi:10.3389/fnana.2022.838567)
Supplement: Supplementary file 1 [file Data_Sheet_1.pdf]

Table. S1. Probes used in the in situ hybridisation staining

Probe name	Reference	NCBI number
dbx1a	Present results	NM_131158.1
gbx2		NM_152964.1
lef1		NM_131426.1
lef1	Dorsky et al., 1999	NM_131426.1
tcf7l2	Li et al., 2009	NM_131259.1
shha	Krauss et al., 1993	NM_131063.3
ascla	Flasse et al., 2013	NM_131219.1
lhx9	Peukert et al., 2011	NM_001017710.2
gad1b	Schollp et al., 2009	NM_194419.1
vglut2.2		NM_001009982.1
nkx2.2a	Corallo et al., 2013	NM_001308640.1
pax6a	Veien et al., 2008	NM_131304.1
pou4f1 (brn3a)	Aizawa et al., 2005	NM_001312866.2
pax7a	Seo et al., 1998	NM_131325.2

Table S2. Primers used in cloning RNA probes for *in situ* hybridisation. Green - sequence designed in IDS, red - restriction side for enzymes, black - additional nucleotide necessary for enzymes attachment.

Gene	Primer sequences
*lef1*_forward	AGAATTCATGCAGCTTTACCCAGGATGGT
*lef1*_reverse	AATGTCGACCTGCAGCCGAGGAGCAGATT
*gbx2*_forward	AGAATTCCAAATCGCGCACGCCTTAAA
*gbx2*_reverse	AATGTCGACCAGCCAAACTGTCACTTGTTTCC
*dbx1a*_forward	AGAATTCTCCAAGGAGAGGGAGTTGCTTT
*dbx1a*_reverse	AATGTCGACGTGGAACCATAACGTGGTTGAACA

Table. S3. Antibodies used in the research and their dilutions. IF – immunofluorescence, WM – whole mount immunostaining

Antibody	Antigen	Host	Dilution	Producer<br>Catalog number	Application
Primary	β-catenin (H-102)	rabbit	1:100	Santa Cruz Biotechnology (sc7199)	IF
	TCF7L2 (6H53)	mouse	1:400 / 1:100	MerckMillipore (05-511)	IF
	Lef1	mouse	1:750	Produced in Prof. Richard Dorsky Lab	IF
	PAX6 (Pax6a/b)	rabbit	1:250	GeneTex (GTX128843)	WM/IF
	anti-Acetylated Tubulin clone 6-11B-1	mouse	1:100	Sigma-Aldrich (T 6793)	WM/IF
	anti-TCF7L2	rabbit	1:100	Cell signalling (C48H11)	IF (mouse)
	anti-PAX6	rabbit	1:200	Biolegend (901301)	IF (mouse)
	anti-GAD1	mouse	1:500	Merck Millipore (MAB5406)	IF (mouse)
Secondary	anti-mouse Alexa 594	goat	1:200 / 1:800 / 1:500 (mouse)	Thermo Fisher (A-21203)	WM/IF
	anti-rabbit Alexa 488	goat	1:200 /1:800 / 1:500 (mouse)	Thermo Fisher (A-21206)	WM/IF

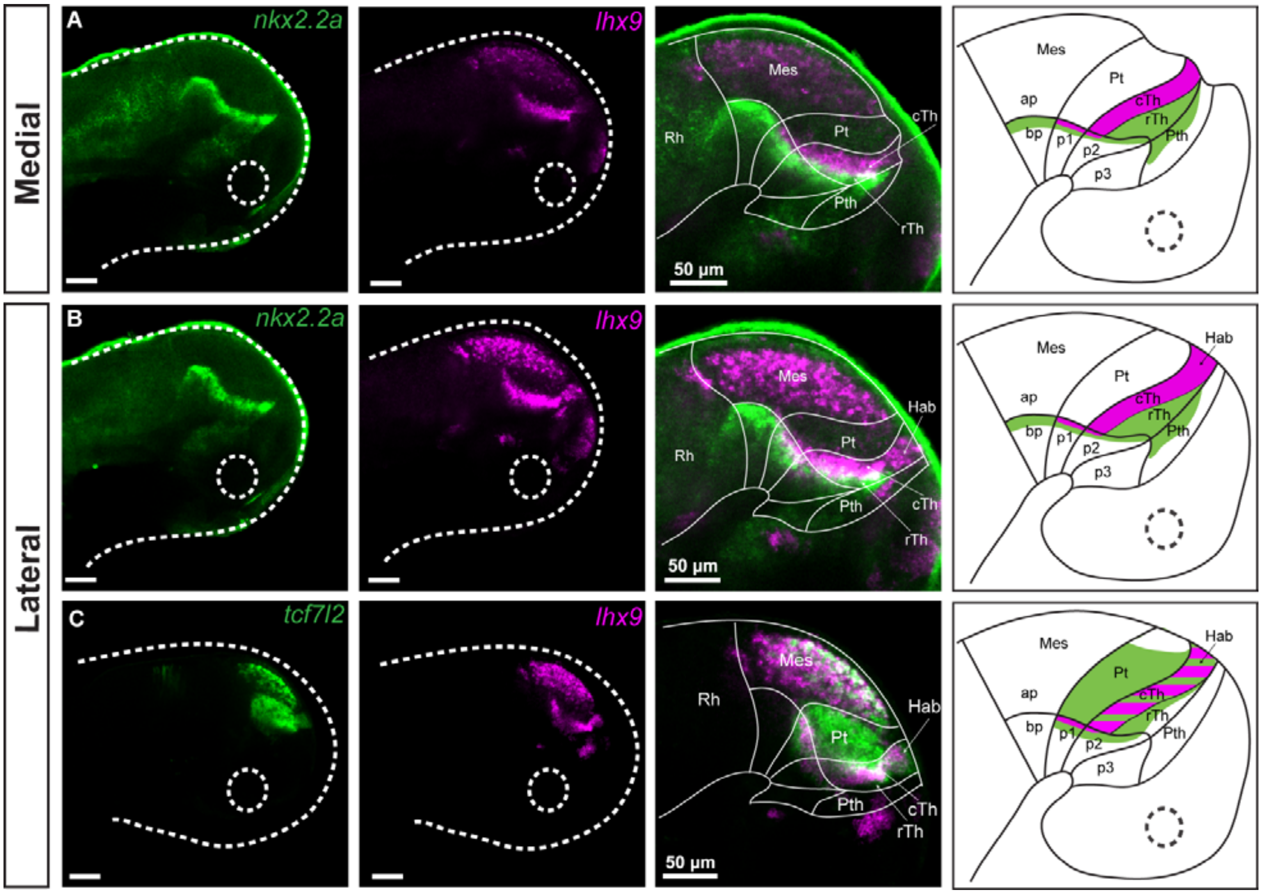

Supplementary Figure 1. Identification of the alar-basal boundary in the diencephalon of 72 hpf zebrafish. Confocal Z-stack images showing brain sections stained using the in situ hybridization with *nkx2.2a*, *lhx9* and *tcf7l2* probes. **(A-B)** Basal plate expression of *nkx2.2a* and *lhx9* delineates the alar-basal boundary in prosomere 1 in medial **(A)** and lateral **(B)** sections. *nkx2.2a* expression also identifies the rTh and Pth, while *lhx9* expression identifies the cTh and Hab (prosomere 2). (**C)** Co-staining of *tcf7l2* and *lhx9* identifies the border between prosomere 1 and 2 in the alar plate. Expression of *tcf7l2* marks the caudal border of the Pt. The schemes show regions identified by the markers in each merged image. Co-expression of *tcf7l2* and *lhx9* in **(C)** is represented by stripe pattern. ap – alar plate, bp – basal plate, Hab - habenula, Mes – mesencephalon, Pt - pretectum, p1 - prosomere 1, p2 - prosomere 2, p3 - prosomere 3, Pth - prethalamus, Rh – rhombencephalon, cTh- caudal thalamus, rTh - rostral thalamus

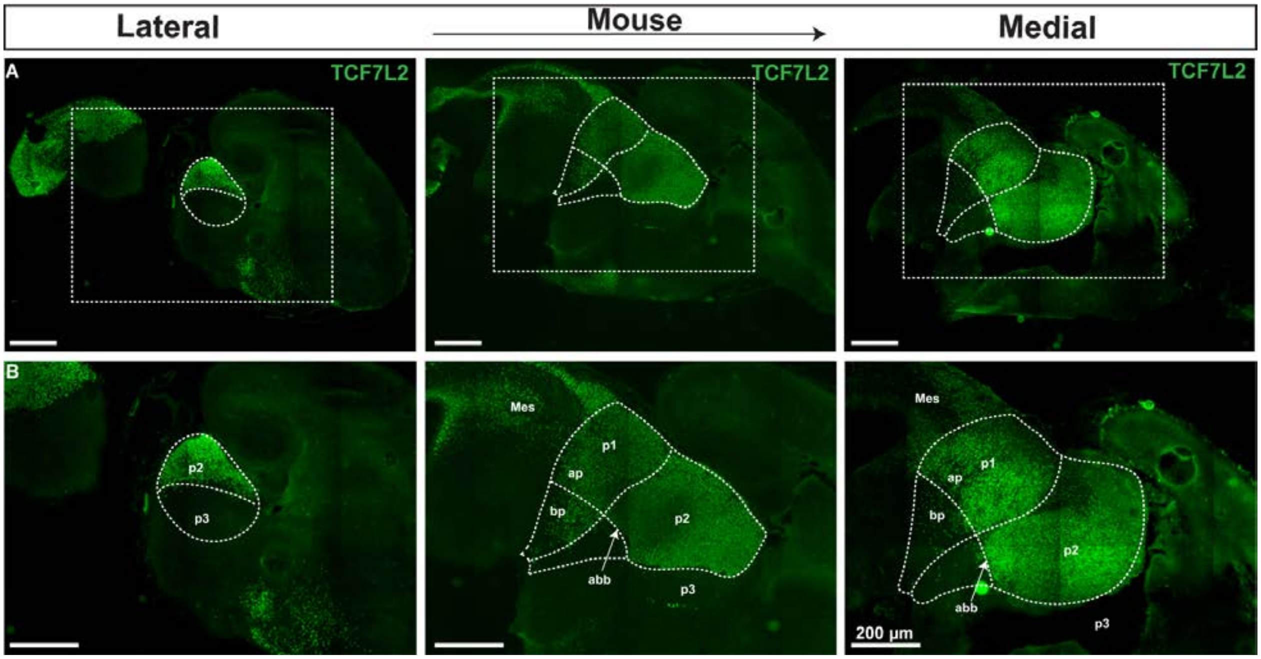

Supplementary Figure 2. Identification of the pretectal boundaries in mouse embryos at E16.5. Images of mouse sagittal brain sections immunostained with an antibody specific for TCF7L2. (**A**) medial to lateral view of the diencephalon shows TCF7L2 in prosomere 1 and 2 alar plate and the basal plate of prosomere 1. **(B)** High magnification images show the boundary between prosomere 2 and 3 in the lateral view and the border between prosomere 1 and 2 in the medial view. abb – alar basa plate boundary, ap – alar plate, bp – basal plate, Mes – mesencephalon, p1 - prosomere 1, p2 - prosomere 2, p3 - prosomere 3

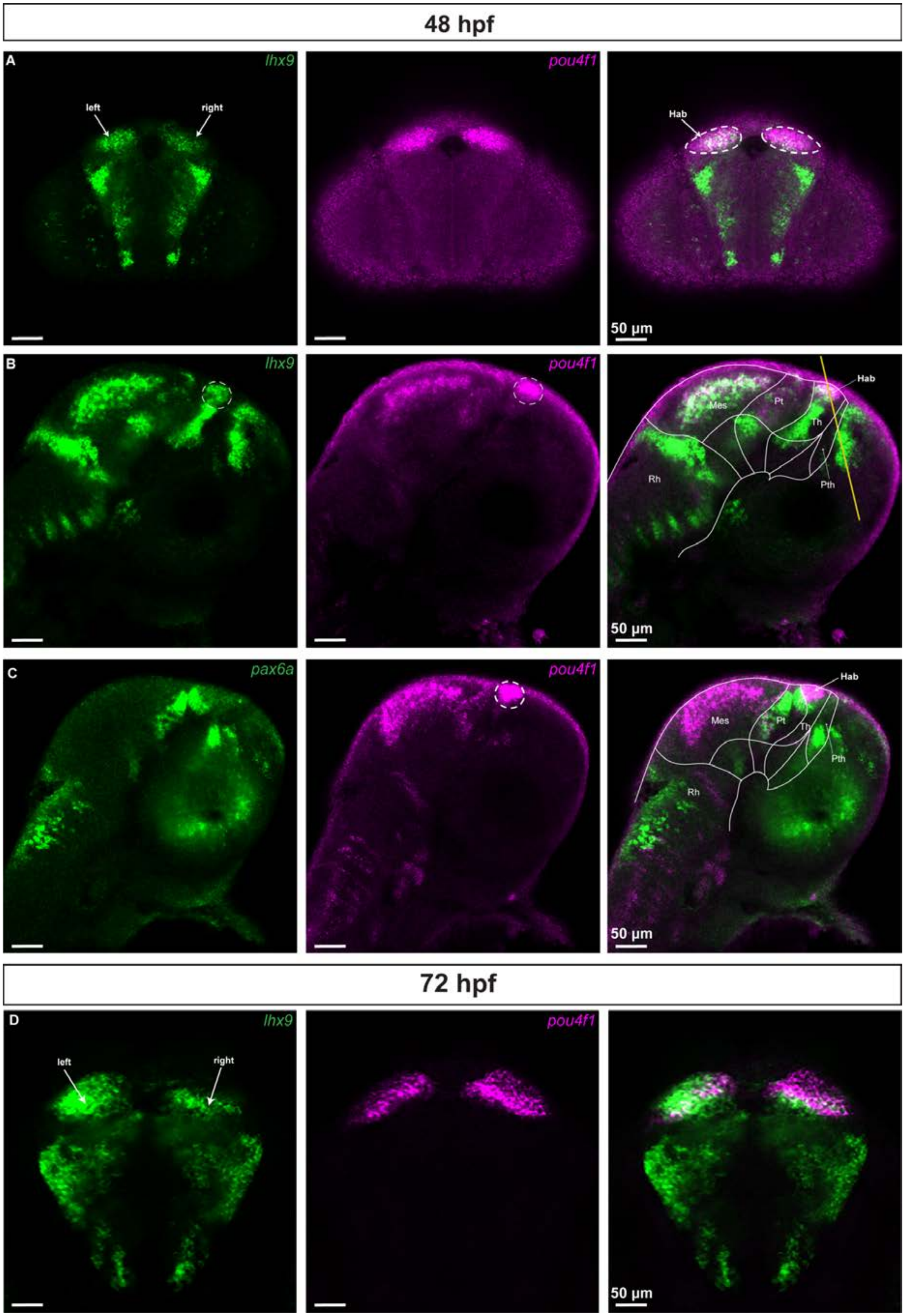

48 hpf
A
lhx9
left
right
pou4f1
Hab
50 µm
B
lhx9
pou4f1
Hab
Mes
Pt
Th
Rh
Pth
50 µm
C
pax6a
pou4f1
Hab
Mes
Pt
Th
Pth
Rh
50 µm
72 hpf
D
lhx9
left
right
pou4f1
50 µm

Supplementary Figure 3. Identification of the habenula in the diencephalon of 48 hpf and 72 hpf zebrafish. Confocal Z-stack images showing brain sections stained using in situ hybridization with *lhx9*, *pou4f1* and *pax6a* probes. **48 hpf:** (**A)** *lhx9* and *pou4f1* co-expression shows the localization of the habenula in the transverse section; in the habenula, *lhx9* is expressed asymmetrically. (**B-C)** Co-staining of *pou4f1* and *lhx9* **(B)** or *pax6a* **(C)** localizes the habenula in the dorsal part of prosomere 2 in the sagittal sections. **72 hpf:** (**D)** *lhx9* and *pou4f1* co-expression shows the localization of the habenula in the transverse section; the expression of *lhx9* in the habenula is strongly asymmetric. The yellow line in (**B)** show the sectioning plane of the transverse sections in (**A)** and (**D)**. Hab - habenula, Th – thalamus, Mes – mesencephalon, Pt - pretectum, Pth - prethalamus, Rh – rhombencephalon

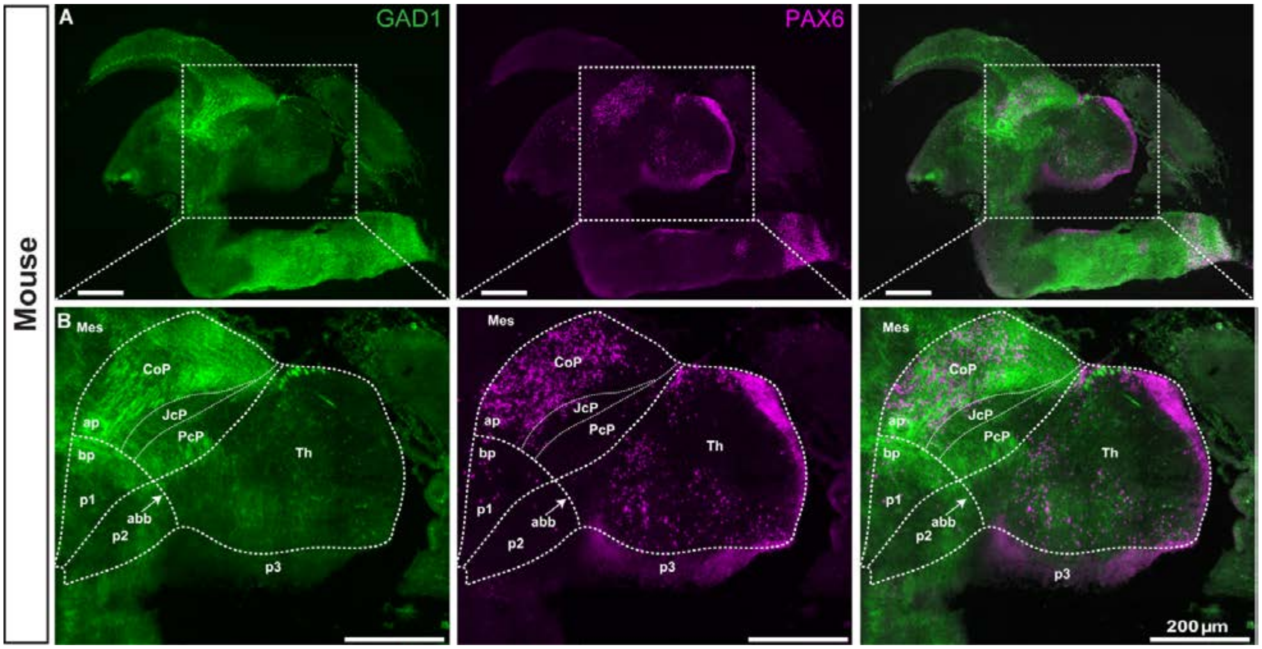

Supplementary Figure 4. Expression of the GAD1 and PAX6 proteins in the mouse pretectum at E16.5. Images of mouse sagittal brain sections immunostained with antibodies specific for GAD1 and PAX6. (**A-B)** PAX6 and GAD1 co-staining identifies the caudal boundary of prosomere 1 and rostral border of the CoP, indicated in high magnification images **(B)**. abb - alar-basal boundary, ap – alar plate, bp – basal plate, CoP - commissural pretectum, JcP - juxtacommissural pretectum, PcP - precommissural pretectum, Mes – mesencephalon, p1 – prosomere 1, p2 – prosomere 2, p3 – prosomere 3, Th – thalamus

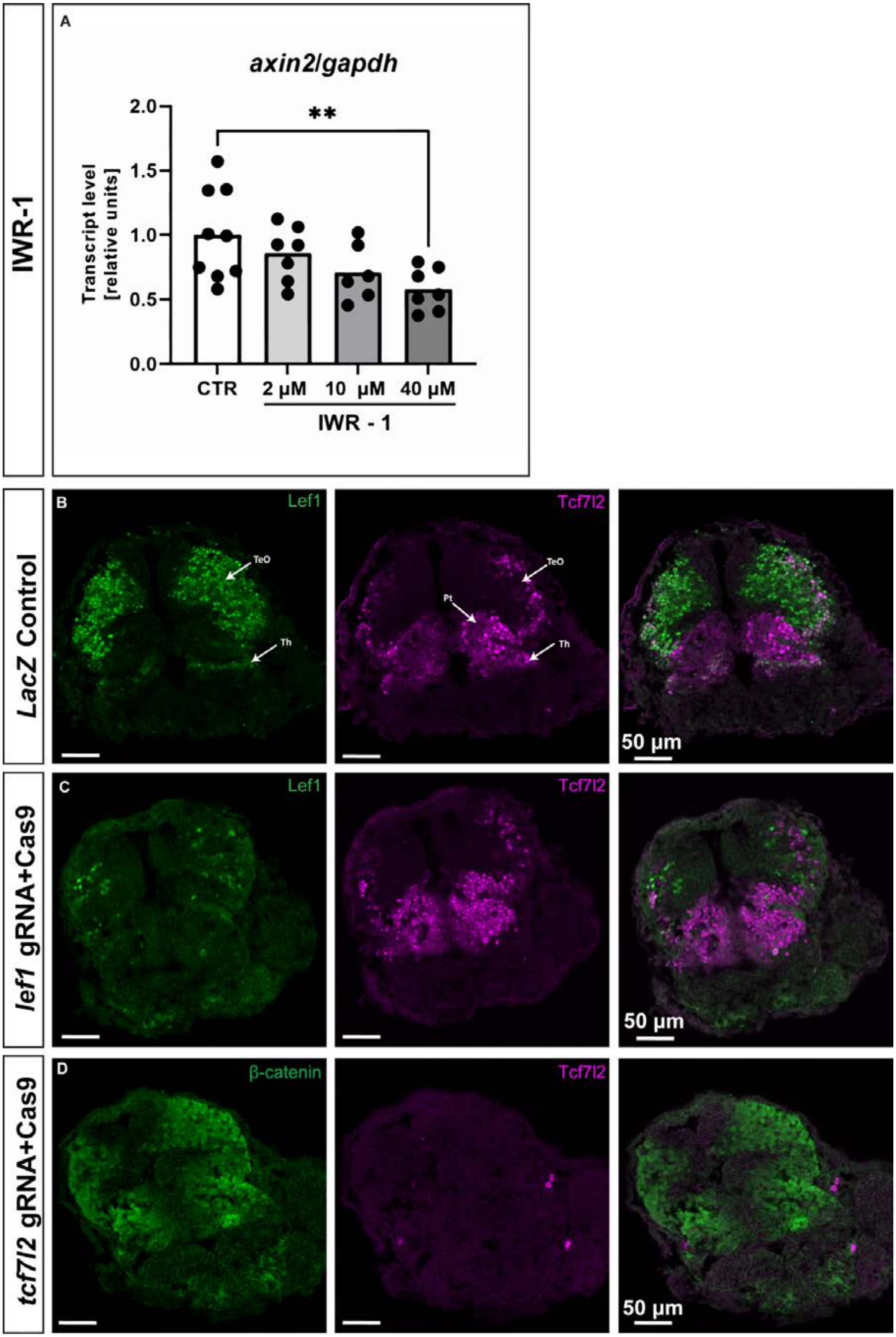
IWR-1
A
axin2/gapdh
Transcript level [relative units]
2.0
1.5
1.0
0.5
0.0
**
CTR
2 µM
10 µM
40 µM
IWR - 1
LacZ Control
B
Lef1
Tcf7l2
TeO
Th
Pt
50 µm
lef1 gRNA+Cas9
C
Lef1
Tcf7l2
50 µm
tcf7l2 gRNA+Cas9
D
β-catenin
Tcf7l2
50 µm

Supplementary Figure 5. Validation of Wnt signalling inhibition by IWR-1 and efficiency of CRISPR/Cas9-mediated knockout of *lef1* and *tcf7l2* in zebrafish at 48 hpf. RT-qPCR analysis of the expression of a classical Wnt pathway target *axin2* in zebrafish embryos threated with IWR-1-between 24 and 48 hpf, and images showing brain sections from F0 crispants immunostained with antibodies specific for Lef1 or Tcf7l2**. (A)** The downregulation of *axin2* indicates that Wnt signalling is inhibited by 24 h treatment with 40 uM IWR-1. (**B-D)** *lacZ* **(B)**, *lef1* **(C)** or *tcf7l2* **(D)** guide RNAs were injected into zebrafish zygotes. Lef1 is almost completely depleted in the brain of *lef1* crispant zebrafish (n=3), and Tcf7l2 is virtually absent in *tcf7l2* crispant zebrafish (n=4). Statistics in (**A)**: p=6-9; one-way ANOVA followed by Dunnett's *post hoc* test; ** $p < 0.01$

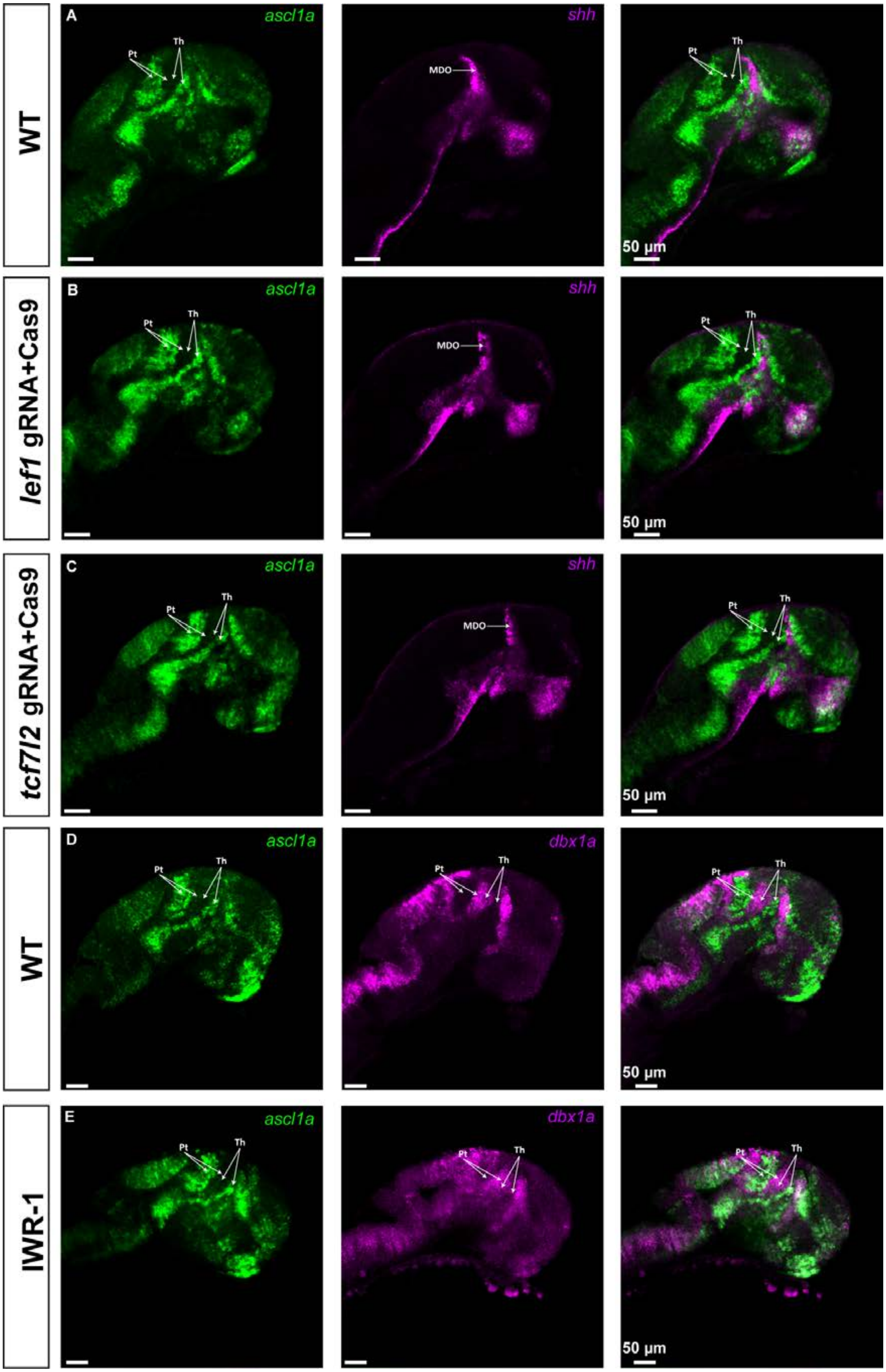
WT
A
ascl1a
shh
Pt
Th
MDO
50 µm
lef1 gRNA+Cas9
B
tcf7l2 gRNA+Cas9
C
D
dbx1a
IWR-1
E

Supplementary Figure 6. Patterning of progenitors in the diencephalon of IWR-1-treated, *lef1* crispant and *tcf7l2* crispant zebrafish at 30 hpf.

Confocal Z-stack images showing brain sections of control, IWR-1-treated (24-30 hpf) and crispant zebrafish embryos stained using in situ hybridization with *ascl1a*, *shh1* and *dbx1a*. (**A-E)** *ascl1a* expression visualizes the pretectal progenitor domain. (**A-C)** *shha* staining identifies MDO. (**D-E)** *dbx1a* expression visualizes the thalamic progenitor domain. The patterning of the diencephalon is not visibly affected by *lef1* **(B)** and *tcf7l2* knockout **(C)** or IWR-1 treatment **(E)** at the stage of 30 hpf. Pt - pretectum, MDO - mid-diencephalic organizer, Th – thalamus

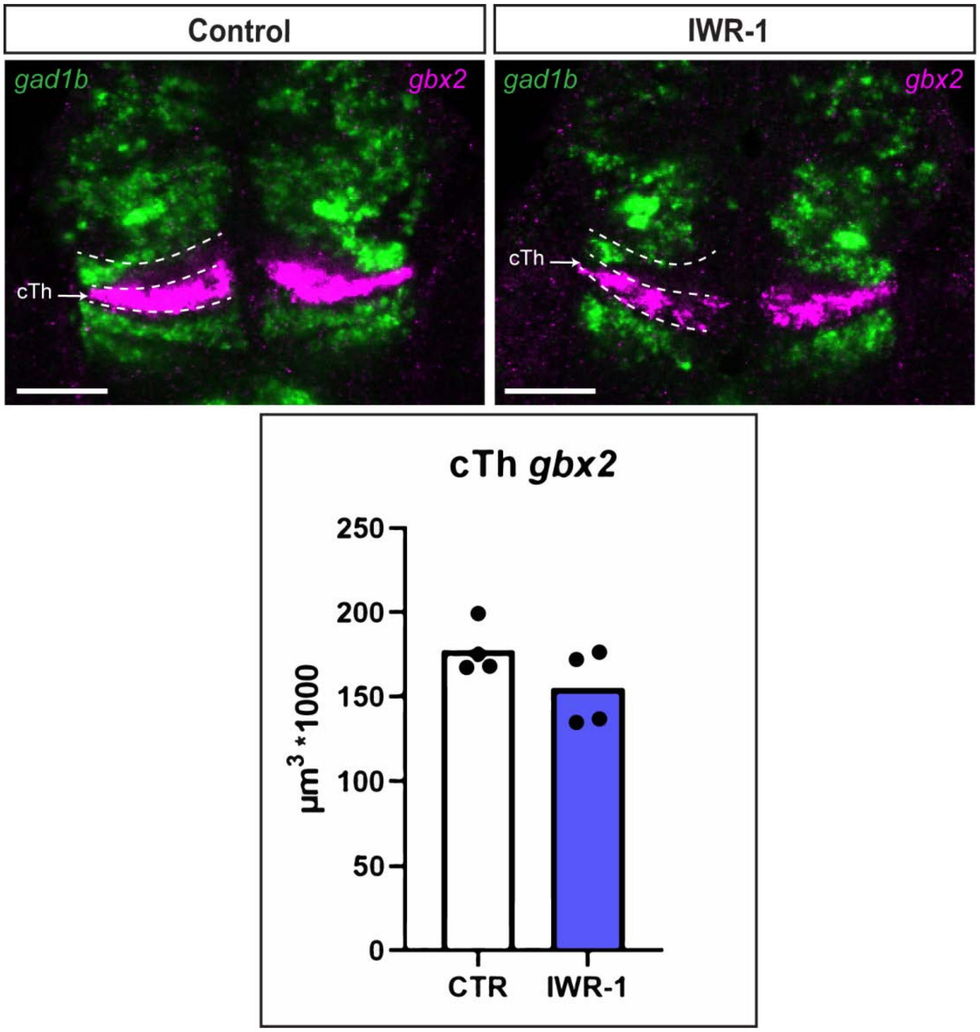

Supplementary Figure 7. Phenotypes in the caudal thalamus and precommissural pretectum of IWR-1-treated zebrafish at 48 hpf. Confocal Z-stack images showing brain sections stained using in situ hybridization with *gad1b* and *gbx2* probes. (**A-B)** The volume of the cTh is not changed by IWR-1-treatement between 24 and 48 hpf, indirectly indicating an expansion of the PcP (compare with Fig. 7). (**C)** The graph shows the volume quantification of cTh indicated in the previous images. Statistics in (C): n = 4; Paired t test. PcP - precommissural pretectum, cTh - caudal thalamus

References

Aizawa, H., Bianco, I. H., Hamaoka, T., Miyashita, T., Uemura, O., Concha, M. L., et al. (2005). Laterotopic representation of left-right information onto the dorso-ventral axis of a zebrafish midbrain target nucleus. *Curr Biol* 15, 238–243. doi:10.1016/j.cub.2005.01.014.

Corallo, D., Schiavinato, A., Trapani, V., Moro, E., Argenton, F., and Bonaldo, P. (2013). Emilin3 is required for notochord sheath integrity and interacts with Scube2 to regulate notochord-derived Hedgehog signals. *Development* 140, 4594–4601. doi:10.1242/dev.094078.

Dorsky, R. I., Snyder, A., Cretekos, C. J., Grunwald, D. J., Geisler, R., Haffter, P., and Raible, D. W. (1999). Maternal and embryonic expression of zebrafish lef1. *Mech Dev* 86(1-2), 147-150. doi:10.1016/s0925-4773(99)00101-x.

Flasse, L. C., Stern, D. G., Pirson, J. L., Manfroid, I., Peers, B., and Voz, M. L. (2013). The bHLH transcription factor Ascl1a is essential for the specification of the intestinal secretory cells and mediates Notch signaling in the zebrafish intestine. *Dev Biol* 376, 187–197. doi:10.1016/j.ydbio.2013.01.011.

Krauss, S., Concordet, J. P. and Ingham, P. W. (1993). A functionally conserved homolog of the Drosophila segment polarity gene hh is expressed in tissues with polarizing activity in zebrafish embryos. *Cell* 75, 1431-1444. doi: 10.1016/0092-8674(93)90628-4.

Li, N., Felber, K., Elks, P., Croucher, P., and Roehl, H. H. (2009). Tracking gene expression during zebrafish osteoblast differentiation. *Dev Dyn* 238, 459–466. doi:10.1002/dvdy.21838.

Peukert, D., Weber, S., Lumsden, A., and Scholpp, S. (2011). Lhx2 and Lhx9 determine neuronal differentiation and compartition in the caudal forebrain by regulating Wnt signaling. *PLoS Biol* 9, e1001218. doi:10.1371/journal.pbio.1001218.

Scholpp, S., Delogu, A., Gilthorpe, J., Peukert, D., Schindler, S., and Lumsden, A. (2009). Her6 regulates the neurogenetic gradient and neuronal identity in the thalamus. *Proc Natl Acad Sci U S A* 106, 19895–19900. doi:10.1073/pnas.0910894106.

Seo, H. C., Saetre, B. O., Håvik, B., Ellingsen, S., and Fjose, A. (1998). The zebrafish Pax3 and Pax7 homologues are highly conserved, encode multiple isoforms and show dynamic segment-like expression in the developing brain. *Mech Dev* 70, 49–63. doi:10.1016/s0925-4773(97)00175-5.

Veien, E. S., Rosenthal, J. S., Kruse-Bend, R. C., Chien, C. Bin, and Dorsky, R. I. (2008). Canonical Wnt signaling is required for the maintenance of dorsal retinal identity. *Development* 135, 4101–4111. doi:10.1242/dev.027367.
